# Dissecting the function of the DNMT2-homolog (DNMA) in *Dictyostelium discoideum*

**DOI:** 10.1093/g3journal/jkaf152

**Published:** 2025-07-04

**Authors:** Zaza Gelashvili, Denis A Larochelle, Jacqueline M Dresch, Maddison Hincher, Javier F Tabima, Robert A Drewell

**Affiliations:** Biology Department, Clark University, 950 Main Street, Worcester, MA 01610, United States; Cell Biology Department, Memorial Sloan Kettering Cancer Center, 1275 York Avenue, New York, NY 10065, United States; Louis V. Gerstner, Jr. Graduate School of Biomedical Sciences, Memorial Sloan Kettering Cancer Center, New York, NY 10065, United States; Biology Department, Clark University, 950 Main Street, Worcester, MA 01610, United States; Biology Department, Clark University, 950 Main Street, Worcester, MA 01610, United States; Biology Department, Clark University, 950 Main Street, Worcester, MA 01610, United States; Biology Department, Clark University, 950 Main Street, Worcester, MA 01610, United States; Biology Department, Clark University, 950 Main Street, Worcester, MA 01610, United States

**Keywords:** DNMA, RNA methylation, *Dictyostelium*, transcriptome, epigenetics, DNA methylation

## Abstract

Methylation of cytosine residues in nucleic acids plays a critical role in a range of biological activities in eukaryotes, including regulation of transcription, organization of chromatin structure, modulation of translation, cellular differentiation, and development. While much of the scientific focus in this field was centered on DNA methylation over the past few decades, it has also become clear that methylation of RNA is a crucial modification. A group of homologous DNMT2 methyltransferase enzymes in different model organisms are now known to catalyze the transfer of a methyl group to the cytosine at position 38 in tRNA^Asp^_GUC_ molecules. The important biological role for tRNA methyltransferases is highlighted by the fact that the genomes of some model eukaryotes, including *Dictyostelium discoideum, Drosophila melanogaster, Entamoeba histolytica,* and *Schizosaccharomyces pombe,* possess a DNMT2 homolog but do not encode any other enzymes of the DNMT family. In this study, we explore the function of the DNMT2 homolog (DNMA) in *D. discoideum* by examining the phenotypic effects resulting from deletion of this enzyme. Pleiotropic impacts on cell growth, morphology and motility, nuclear organization, and disruption to the developmental program are detected. We also analyze global gene expression in the *dnmA* knock-out cells and develop a homology-based structural model of DNMA, allowing us to perform docking simulations of the molecular interaction with tRNA^Asp^_GUC_. Our findings demonstrate that DNMA, as a tRNA methyltransferase, is critical to normal cellular activity and development in *Dictyostelium*.

## Introduction

DNMT2 orthologs were originally identified in a wide range of species as DNA methylating enzymes, based on their extensive sequence similarity with other proteins in the DNA methyltransferase (DNMT) family. The DNMT1 and DNMT3 homologs are known to catalyze the addition of a methyl (CH_3_) group at the carbon 5 position in cytidine, producing 5-methylcytosine (abbreviated as 5mC in the DNA modification field) (Bestor 2000; Goll and Bestor 2005; Chedin 2011). However, rigorous experimental testing of DNMT2 enzymes revealed that they lack robust DNA methylation activity, but rather are tRNA methyltransferases that catalyze the methylation of position 38 in tRNA^Asp^_GUC_ to generate a 5-methylcytosine (abbreviated as m^5^C in the RNA modification field) (Goll *et al.* 2006). In mammals, the loss of DNMT2 results in a reduction in the steady-state levels of unmethylated tRNAs (Tuorto *et al.* 2012) and the charging of tRNA^Asp^_GUC_ resulting in significantly reduced rates of translation for poly-Asp proteins (Shanmugam *et al.* 2015). While loss of DNMT2 in many model organisms does not result in strong developmental phenotypes under standard laboratory conditions (Jeltsch *et al.* 2017), tRNA fragmentation and reduced viability is detected under heat and oxidative stress conditions (Schaefer *et al.* 2010; Jeltsch *et al.* 2017).

**Fig. 1.**
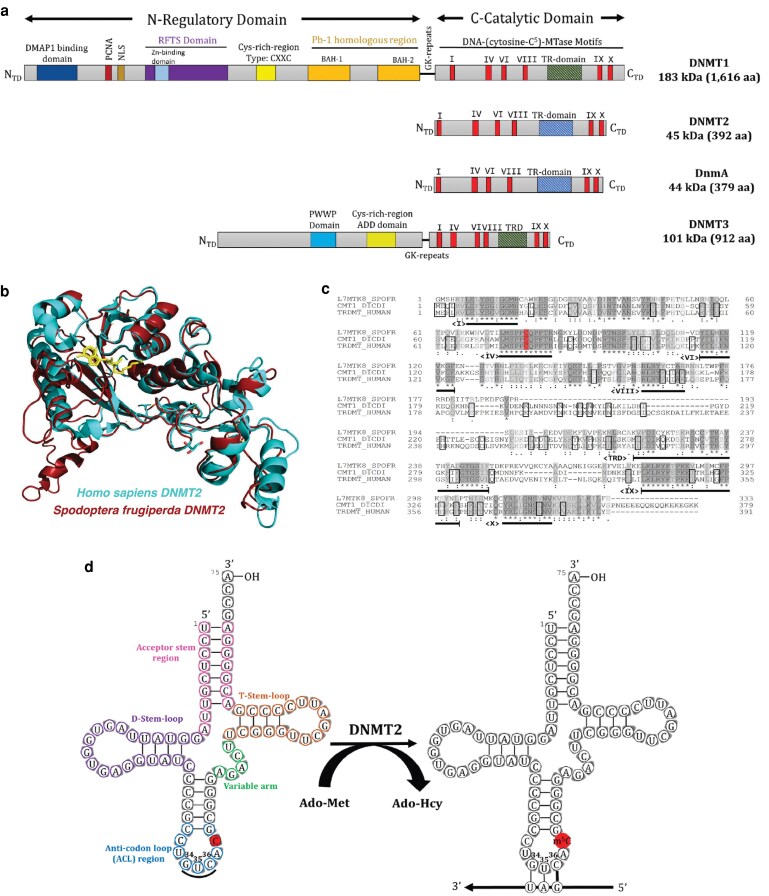
Structure, sequence alignment and function of DNMT2 homologs. a) Conserved domain motifs in eukaryotic nucleic acid methyltransferases. Functional domain organization, molecular weight, and number of amino acid residues in human DNMT1, DNMT2, DNMT3(A), and *D. discoideum* DNMA are shown. All the proteins share the conserved C terminal-catalytic DNA-(cytosine-C^5^)-methyltransferase domain, including the highly conserved motifs I, IV, VI, VIII, IX and X (bright red). DNMT2 and DNMA lack the additional N-terminal domains found in DNMT1 and DNMT3, but do contain the TR-domain (hashed blue) characteristic of the DNMT2 protein family. DMAP1, DNMT1-associated transcription repressor domain (dark blue); PCNA, proliferating cell nuclear antigen binding domain (dark red); NLS, nuclear localization sequence (dark orange); RFTS, replication foci targeting sequence (purple); CXXC, cysteine rich zinc finger DNA-binding motif (yellow); BAH, bromo-associated homology domains (light orange); GK repeats, lysine–glycine repeats (black); PWWP, conserved proline–tryptophan–tryptophan–proline motif (solid blue). b) Structural alignment of DNMT2 homologs. Superposition of X-ray crystal structures of human DNMT2 (PDB ID: 1g55, cyan) and *Spodoptera frugiperda* DNMT2 (PDB ID: 4h0n, red). The Ado-Hcy co-factor is highlighted in yellow. c) Sequence alignments of DNMT2 proteins. FASTA amino acid sequence alignment of the DNMT2 homologs from *Spodoptera frugiperda* (L7MTK8_SPOFR), *Dictyostelium discoideum* (CMT1_DICDI) and human (TRDNMT_HUMAN) are shown. Dashed lines indicate sequences that are missing from the available ESTs. Highlighted amino acids are either invariant (dark gray), high similarity (gray) or low similarity (light gray). Regions corresponding to the catalytic motifs (I, IV, VI, VIII, IX and X) are labeled below the sequence alignment. d) tRNA cloverleaf structure with cytosine 38 highlighted as the target of DNMT2 enzyme methylation. DNMT2 utilizes the methyltransferase catalytic mechanism to transfer a methyl group from the Ado-Met co-factor donor to C^38^ in the tRNA^Asp^_GUC_ substrate molecular, resulting in m^5^C at that nucleotide position (red circle) and yielding Ado-Hcy as a byproduct.

In the social amoeba *Dictyostelium discoideum*, the DNMT2 homolog is named DNMA and it is the only member of the DNMT family present in this species (Goll and Bestor 2005; Jeltsch *et al.* 2017). DNMA shares sequence conservation of essential catalytic motifs and exhibits sequence similarity to other DNMTs in a wide range of species (Fig. 1a–c) (Dong *et al.* 2001; Schaefer and Lyko 2010; Jurkowski and Jeltsch 2011; Jeltsch *et al.* 2017; Wadhwa *et al.* 2024). Prior studies have confirmed that DNMA predominantly methylates tRNAs, with the tRNA^Asp^_GUC_ as the preferred target substrate, both *in vivo* and *in vitro* (Fig. 1d) (Muller *et al.* 2013) and that, as might be expected in an organism lacking DNMT1 and DNMT3, there is no biologically significant DNA methylation in *D. discoideum* (Drewell *et al.* 2023). A genetic knock-out (KO) of *dnmA* uncovered a functional role in silencing retrotransposons (Kuhlmann *et al.* 2005) and developmental defects, including fragmentation of the sori along the stalk of the fruiting body during culmination and inefficient sporulation (Katoh *et al.* 2006).

As a result of these observations, some key questions have emerged. What are the underlying molecular and biological consequences of DNMA-mediated RNA methylation and how do these impact cellular function, organization, and development? Is the structure of the DNMA protein similar to the other members of the DNMT2 group and could this shed light on its functional activity? Is tRNA methylation the sole activity of DNMA or could there be some level of promiscuity in substrate recognition? In this study, we directly investigate the biological impact of the loss of enzymatic activity in cells lacking DNMA. Comparison to wild-type (WT) cells enabled us to characterize a wide range of phenotypic effects associated with deletion of the *dnmA* gene including: cell proliferation, cell size and morphology, nuclear and centrosome organization, cell motility, and disruption to the developmental program. We also analyzed the transcriptome in KO and WT cells to gain insight into changes in gene expression across the entire genome. In addition, we leveraged experimentally verified crystal structures of DNMT2s from other species to develop a homology-based model of DNMA structure and performed docking simulations to demonstrate its molecular interaction with the tRNA^Asp^_GUC_ substrate.

## Materials and methods

### Sequence analysis of DNMTs

The primary amino acid FASTA sequence of DNMA (DDB_G0288047) was initially retrieved from dictyBase (Fey *et al.* 2006). This sequence was used to identify mammalian DNMTs, *Dictyostelium* DNMA (*D. discoideum* DNMA, Q54JH6, 44.1 kDa) and additional members of the DNMT2 family of proteins from other species: *Homo sapiens* TRDMT1 (O14717, 44.6 kDa) and *Spodoptera frugiperda* TRDMT1 (L7MTK8, 38.4 kDa) in the UniProt database. The corresponding atomic coordinate data files for the resolved crystal structures were retrieved for the *Homo sapiens* (hDNMT2, ID: 1g55) (Dong *et al.* 2001) and *Spodoptera frugiperda* (SfDNMT2, ID: 4h0n) (S. Li *et al.* 2013) DNMT2 proteins through the RCSB PDB database (Berman *et al.* 2000). The amino acid sequences of DNMA, hDNMT2 and SfDNMT2 were compared using multiple pairwise alignments in ClustalW Omega (The UniProt Consortium 2024).

### Cell culture, growth curves and size measurements

*D. discoideum* AX4 WT and AX4 *dnmA* KO cells were grown in HL5 liquid cultures (Formedium Ltd, Hunstanton, UK) and routinely passaged to ensure their density never exceeded 5 × 10^6^ cells/ml. Stationary cultures were grown in 100 × 15 mm sterile polystyrene Petri dishes at room temperature. Shaking cultures were grown in 75-mL Erlenmeyer flasks on an orbital shaker at constant 200 rpm. Developing cells were obtained via transfer to starvation buffer as previously described (Drewell *et al.* 2023).

Growth curves were generated by inoculating cells at a starting concentration of 1 × 10^5^ cells/mL in either stationary or shaking conditions. The cultures were set up in replicates of three and the assay was repeated a total of three times, with each experiment conducted on freshly thawed batches of WT and KO cells. Cell counts were recorded every 24 h, for 6 d (144 h) using an automated cell counter (Cellometer Auto T4—Nexcelom Bioscience) by extracting 20 μL from the shaking cultures and resuspended stationary cultures. The cell concentrations and mean diameter (μm) were automatically calculated using default Cellometer parameters. Growth curves were calculated using the average values best fit to a simple logistic function. The cell diameter values were subjected to two statistical tests of normality of distribution: The one-sample Kolmogorov–Smirnov test (KS Test) comparing the cumulative distribution function of cell diameters to that of a standard normal distribution and Pearson's chi-square (χ^2^) test. The statistical tests were performed in MATLAB, with significance defined as a *P* value of <0.05.

### Confirmation of *dnmA* KO

AX4 strain cells carrying a KO of the *dnmA* gene were generated by Katoh *et al.* (2006) and generously provided by Gad Shaulsky. The cells were maintained as described in previous section. Fresh cells were routinely thawed on a monthly basis from frozen spores to ensure low passage number cells were used for all analysis and protect against potential genetic drift. To confirm the absence of the *dnmA* gene in the KO cells, we determined the read depth across the gene (chromosome 5: 1026957–1028573) in AX4 KO and WT cells in our previously published whole genome sequencing datasets (Drewell *et al.* 2023). We observed a sharp reduction in read depth across the *dnmA* gene, with a median coverage of 1× compared with 117× across the entirety of chromosome 5. In contrast, in the WT strain there was consistent coverage across the *dnmA* gene (83×) and entire chromosome (97×). As a control, read depth at the *trmt5* gene (chromosome 3: 2486411–2488090) was found to be consistent between WT and KO strains, confirming the specificity of the *dnmA* deletion (Supplementary Fig. 1) (Drewell *et al.* 2023).

### Fluorescence microscopy

Cells were cultured as described in the previous section and transferred to 6-well plates. Once cells were attached, as determined by microscopic observations, they were fixed as follows. An initial pre-fixation step was carried out by adding 2 mL ice-cold 100% methanol to the 2 mL HL5 culture media present in each well. The media/methanol mixture was then replaced with 2 mL 100% ice-cold methanol and the plate immediately placed at −20 °C for 10 min for fixation. This step was followed by 3 to 5 washes with room temperature PBS (137 mM NaCl, 2.7 mM KCl, 10 mM Na2HPO4, pH = 7.4) for 5 min each wash.

Fixed cells were either stained with DAPI to visualize the nuclei and/or with 1 or 2 protein-specific antibodies. The antibodies used in this study were: rabbit anti-DdSun1 (I. Schultz, O. Bauman, C. Zoglmeier, M. Samereier, and R. Gräf) (1:50 dilution), mouse anti-α-tubulin (12G10, Developmental Studies Hybridoma Bank, Iowa U.S.A.) (1:100 dilution), rabbit anti-DdNE81 (provided by Dr. Ralph Gräf) (1:1,000 dilution), and mouse anti-DdCP224 (provided by Dr. Ralph Gräf) (1:2 dilution). For staining solely with DAPI, fixed and washed cells were incubated with 2 μg/mL DAPI (Sigma-Aldrich D9542) in PBS for 10 min, followed by two washes in PBS. For immunofluorescence staining all initial antibody incubations were preceded by blocking with 5% goat serum diluted in PBST ((137 mM NaCl, 2.7 mM KCl, 10 mM Na2HPO4, pH = 7.4) for 30 min. Primary antibody incubations were carried out with antibodies diluted to the appropriate concentration in PBST and allowed to bind for 60 min at room temperature. This was followed by three washes in PBST. Similarly, secondary antibody incubations were conducted as the primary antibody incubations, but for 90 min. Secondary antibodies used in this study were goat anti-mouse or anti-rabbit antibodies tagged with either Alexa Fluor 488, Alexa Fluor 555, or Alexa Fluor 568 (ThermoFisher-Invitrogen).

For double labeling with antibodies the above protocol was essentially followed (typically modified by increasing the concentration and incubation times for both the primary and secondary antibodies). Following labeling with the first primary and secondary antibody sequence the cells were washed five times in PBST before the procedure was repeated with the second primary and secondary antibodies. For cells double labeled with antibodies and DAPI, the DAPI staining, as described above, was carried out following the antibody labeling.

Cells were imaged on a Nikon Eclipse E600 upright widefield fluorescence microscope under 100× objective lens. The images were acquired at 6.45 µm pixel size at 8-bit intensity resolution with a 24 × 24 µm field of view and processed using SPOT basic software (SPOT imaging). Confocal images were obtained on a Leica TCS SP5 laser-scanning microscope. The images were acquired in XYZ scan mode, where X and Y correspond to 71.5 × 71.5 µm field of view (1,024 × 1,024 pixels) and approximately 20 to 30 z-step confocal slice sections were acquired at step size 0.29 µm for a total depth of ∼8 µm (Voxel: 69.9 × 69.9 × 293.7 nm XYZ) The cell Images were acquired with 63× objective with 3.4 digital magnification (SCANx), line-averaging = 3, scan-speed = 200, frame-average = 1 and resolved with native 8-bit image intensity resolution. Confocal images were processed using LAS AF (v. 3.1.0) for further analysis.

### Live development and two-dimensional cell tracking analysis

To compare AX4 WT and *dnmA* KO cells, we initiated starvation and observed the live progression of development and culmination over 48 h. Cells were starved as previously described (Drewell *et al.* 2023) and the resulting cell suspension was plated on a gridded nitrocellulose membrane (Whatman). Time-lapse recording was obtained by live filming development for 48 h. A total of four replicates of the live development recording experiments were conducted with WT and KO cells. For the time-lapse analysis, the obtained raw video data files were processed through two independent pipelines. Initially, the 48 h-long movies were compressed, and play-time speed was accelerated 400× using OpenShot Software, to allow compression of 48 hour. long movies into multiple shorter 16 to 18 s development recordings without the loss of any frames. Additionally, raw images with a period of 3 min were collected from the entire recordings of the four independent replicates, compiled and transformed to defined X–Y–T [two-dimensional (2D) space over time] pixel coordinates for data extraction in FIJI (Image J, NIH), which enabled the inversion of colors and adjustment for contrast and brightness values, and Imaris 9.5.5 (Bitplane, Oxford Instruments).

The processed video image files were then analyzed using a tracking algorithm in Imaris 9.5.5 (Bitplane, Oxford Instruments). In brief, the 2D recordings of development were initially analyzed using a total cost function algorithm that allowed us to find the best track connections. The model was then further fit into a defined compute-cost based function, using autoregressive motion that captures the characteristic spiraling and oscillatory movements observed in *Dictyostelium*. The autoregressive motion model, AR1, best fits the data, as it applies speed constrains and computes direction, distance, and motion (Bitplane ). Maximum track length distances for individual motile cells and slugs were defined automatically from the video utilizing a gap-closing algorithm to create tracks by linearly connecting associated objects within the vicinity of the same track. To capture the later stages of development, mounds were recreated in two dimensions using identified spots and were tracked over time within a defined pixel intensity change of the recording. Once fruiting body formation initiated, the spot voxels were allowed to grow in order to trace the movement from the aggregated mounds to the elongated stalk cells that ultimately support a sorus of spores. All track surfaces were rendered to illustrate the movement of developing cells over time. Through the Imaris software, we were able to extract relative speed, motion and track length as numerical values (Bitplane ). These data were further processed through RStudio for graphical display using *ggplot2*.

### Transcriptome analysis

A total of four biological replicates each of *D. discoideum* AX4 WT and *dnmA* KO cells were cultured under identical conditions as described in the cell culturing protocol. After 1 wk of growth, cells were harvested via centrifugation, and RNA was extracted using the Direct-zol RNA Miniprep Kit (Zymo Research Corporation, California, USA), following the manufacturer's instructions. RNA quality and integrity were assessed prior to sequencing using agarose gels and by NovoGene Corporation. Library preparation for RNA sequencing was performed by NovoGene Corporation, employing poly(A) mRNA enrichment and sequencing on an Illumina NovaSeq platform to generate 150 bp paired-end reads.

Sequenced mRNA reads were mapped to the *D. discoideum* AX4 reference genome (Eichinger *et al.* 2005) using HISAT2 (Zhang *et al.* 2021). Gene-level read counts were quantified using *htseq-count* from the HTSeq package (Anders *et al.* 2015). Differential gene expression analysis was conducted in the R package DESeq2 (Love *et al.* 2014), comparing WT and *dnmA* KO samples to identify genes with statistically significant changes in expression levels. Counts were normalized using the DESeq2 median-of-ratios method, and genes with fewer than 10 total counts across all samples were excluded. Differentially expressed genes were identified based on a log2 fold-change threshold of ±2 and an adjusted *P*-value (FDR) < 0.1 using the Benjamini–Hochberg correction. Principal component analysis (PCA) was conducted to visualize sample relationships. Volcano plots, heatmaps and boxplots used to compare the differentially expressed genes were constructed using ggplot2 (Wickham 2016).

### Protein structural analysis

The Expasy ProtParam server (Gasteiger *et al.* 2005) was used to compare the physiochemical characteristics of DNMT2 proteins, including theoretical isoelectric point (pI), molecular weight, the total number of polar (positive and negative) residues, and instability index. A scan for possible disulfide bond (S–S) predictions was performed using the GDAP-omicX program (O'Connor and Yeates 2004). Secondary structure analysis was performed by classification of protein folds from the similarity of their structure and corresponding primary amino acid sequence. The structures were analyzed using two methods of structural classification, SCOPe (Structural Classification of Proteins—extended) and CATH (Class/Architecture/Topology/Homologous-Superfamily) (Csaba *et al.* 2009).

Atomic coordinate data files were retrieved through the RCSB PDB database (Berman *et al.* 2000) for three DNMT2s with a resolved crystal structure: *Homo sapiens* (hDNMT2, ID: 1g55) (Dong *et al.* 2001), *Spodoptera frugiperda* (SfDNMT2, ID: 4h0n) (S. Li *et al.* 2013) and *Entamoeba histolytica* (EhMETH, ID: 3qv2) (E. C. Schulz *et al.* 2012). The structures were analyzed using Molecular Operating Environment (MOE 2018.01.01) software to enable the visualization, modeling, and simulation of the DNMT2 proteins. The X-ray coordinate files were adjusted for hydrogen atoms using the Protonate 3D tool (*T* = 310 K, pH = 7.4, [salt] = 0 mol/L, Electrostatics = GB/VI, Dielectric = 4, VDW = 800R3), and subjected to algorithm SVL command that allowed determination of protein secondary structure distribution in the resulting crystal structures. Empirical distribution of coordinate structures were subjected to validation and verification by the explicit generation of general Ramachandran plots. Validation was performed to determine stereochemical aspects along the protein's main backbone and side chains. Overall, all residues in the PDB files were plotted based on their ϕ/ψ ratio, which allows various residues of the DNMT2 proteins to fall under either core, allowed or disallowed regions of the Ramachandran plot.

### Protein homology comparison

A structural model for the *D. discoideum* DNMA protein was generated using the SWISS-MODEL comparative homology modeling approach (Waterhouse *et al.* 2018). The three-dimensional (3D) Cartesian coordinates of the DNMA protein structure were generated by extrapolating experimental information from evolutionarily related proteins. Potential protein templates were initially identified using multiple pairwise alignments of DNMT2 sequences in ClustalW Omega (The UniProt Consortium 2024). The two proteins with the highest similarity were hDNMT2 and SfDNMT2. From these template proteins, a 3D protein model of DNMA was generated by transferring conserved atom coordinates as defined by amino acid residue alignment. The DNMA structure obtained was quantified for modeling errors, according to estimates of expected model inaccuracy, with QMEAN used as a primary mode of reference to adjust distance constraints of the structure (Waterhouse *et al.* 2018). The resulting 3D structure of DNMA was subjected to molecular dynamics under selected forcefield for elucidating global minima which were followed by secondary structural validation and verification by general Ramachandran plots. The structure was refined using solvation, and C-β deviations were adjusted by partial protonation and energy minimization. A united atom forcefield parameter for crystallographic refinement of proteins was used as an energy function to define the potential energy of the whole protein when visualizing the predicted DNMA structure (Musil *et al.* 1991). Finally, the structural alignment was adjusted by considering the location of insertions/deletions in loops when comparing the primary sequence of DNMA and the template DNMT2 proteins. The predicted structure of DNMA was validated via alphafold2 (AFDB/Foldseek, AF ID: AF-Q54JH6-F1-v4) by direct comparison to SfDNMT2, maintaining the predicted model with robust average local residue confidence (pLDDT = 94.71).

### Electrostatic potential maps

The DNMA, hDNMT2 and SfDNMT2 structures were subjected to vacuum electrostatic potential determination. To calculate surface contact potentials, the *Adaptive Poisson–Boltzmann Solver* (APBS-pdb2pqr) plugin was used in PyMOL 2.0 (Schrodinger). The visualization software generated a qualitative electrostatic representation for individual residues using the quasi-coulombic-shaped convolution function to allow the quantification of contact potentials by representing the color-coded surface of proteins in *K*_b_T/e_c_. The potential displayed by the default coloring includes point-charge residue interactions with the solvent.

### Molecular dynamics and protein-tRNA docking simulations

For molecular dynamic simulations, all coordinates in the DNMA, hDNMT2 and SfDNMT2 protein structures were assigned a specific potential energy value of the forcefield and parameters and restraints were adjusted based on previous approaches for crystallographic structures (Musil *et al.* 1991; Olson 2018). The partial charges were used to prepare structures by adjusting hydrogens and lone pairs as defined by the energy function forcefield. Following the partial charge assignments to individual residues, the structures were first subjected to energy minimization and geometry optimization to locate relatively stable molecular conformations. The structural model allowed the optimization and capture of local minima of total potential molecular energy (*E* = kcal/mol) and the dynamic simulations were conducted to capture the global minima of the total molecular energy of the proteins. Clashes and rotamers with unfavorable total molecular energy conformation were removed, while keeping all non-hydrogen atoms as close as possible to their starting position. The dynamic conformation of the proteins was analyzed under different temperature or pressure conditions (Hospital *et al.* 2015). The parameters were interpolated during the simulation stage in which the system was heated from 0 to 310 K, accompanied by pressure equilibration at 500 ps. The system was subsequently cooled down from 310 to 0 K with pressure reduced to 100 ps. In total, 100 models were generated by the dynamic simulation algorithm for each selected protein structure. The best model for each of the three proteins was identified based on the individual dynamic simulation and lowest total potential energy value (*E* = kcal/mol).

The crystal structure of tRNA^Asp^ was retrieved from the RCSB PDB database (PDB ID: 1vtq) (Moras *et al.* 1980). An all-atom forcefield CHARMM27 (MacKerell *et al.* 2000) parametrized for RNA crystal structures was applied to tRNA^Asp^, where the charge dictionary is used along with bond-charge increments for nucleic acids in order to define the interatomic and functional model of the structure. The resulting tRNA^Asp^ structure was adjusted for hydrogen atoms by protonate 3D (*T* = 310 K, pH = 7.4, [salt] = 0 mol/L, Electrostatics = GB/VI, Dielectric = 4, VDW = 800R3), similar to the solvation conditions used for the DNMT proteins. The tRNA structure was subjected to local energy minimization for full atom mode-refinement and local minima potential energy conformation.

The global minima potential energy conformations of DNMA, hDNMT2 and SfDNMT2 in complex with the tRNA^Asp^_GUC_ molecule were analyzed using *in silico* docking experiments using Molecular Operating Environment (MOE 2018.01.01) software. DNMT2-tRNA^Asp^_GUC_ docking simulations were performed using the potential energy forcefield model of AMBER10:EHT, which is an all-atom forcefield specifically parametrized for large protein and nucleic acid structures (Case *et al.* 2005, 2023). The electrostatic interactions were set to a *Cutoff* value of 15 Å and the protein was labeled as an enzyme, with the tRNA^Asp^_GUC_ as a substrate. The candidate active sites in the protein were selected as motif IV and VI. The substrate was subjected to the site-finder tool in MOE and the C38 position in the anti-codon loop (ACL) region of the tRNA was selected as the alpha site. The atoms in the backbone were isolated and replaced with dummy atoms to allow for the identification of the pocket location of the enzyme during docking simulations. Both the active site and dummy atoms were selected as hydrophilic sites. *Placement* was set to “Alpha Triangle”, to allow superposition of substrate atom triplets and triplets of enzyme atoms. *Score* was set to “London ΔG”, to allow scoring function estimates for the free energy of binding between each protein and the tRNA^Asp^_GUC_. Structural *Refinement* was set to “Induced fit”, to allow for structural adaptation between the protein and tRNA upon interaction. *Poses* were set to 1,000, and the output database docked poses were sorted by the final S score (London ΔG) and the lowest S score docking conformations identified. The obtained placement models (pose = 1,000) of docked structures were subjected to structural refinement with generalized born solvation model (GBVI/WSA) as an estimate for the free energy of hydration and scored for an additional 100 poses. From the final docked poses the lowest ΔG refined structures were selected to convert the free energy values into dissociation constants using Δ*G* = −RT ln(*K_d_*), where *K_d_* = $e{(}_{-RT}^{\Delta G})$.

## Results

### Cell proliferation and size impacted in DNMA KO

**Fig. 2.**
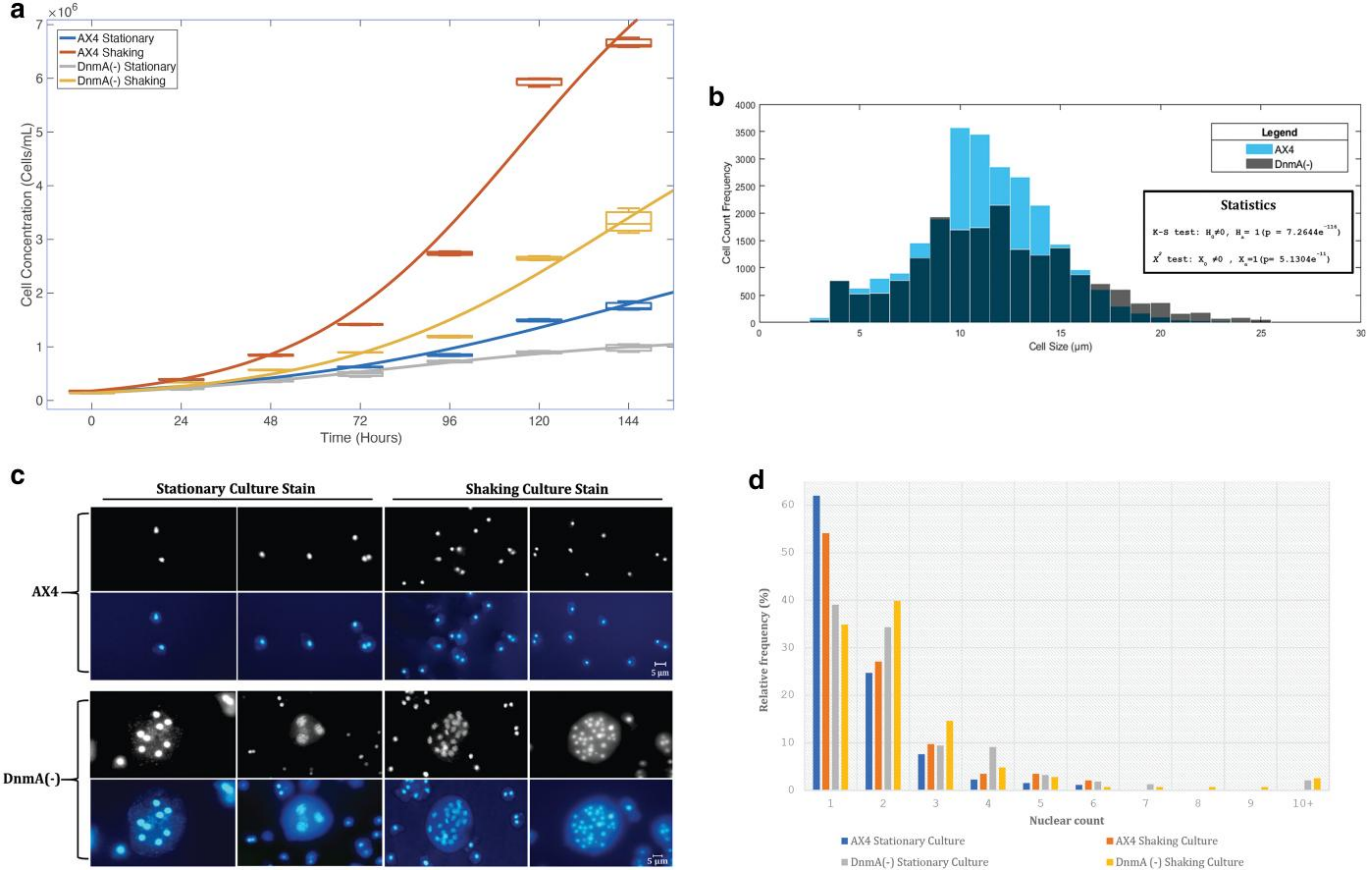
a) The impact of DNMA disruption on cell proliferation. Growth curves for wild-type (AX4) and *dnmA* knock-out (DnmA-) vegetative cells demonstrate that proliferation is lower for the KO cells in both stationary and shaking cultures. The cell cultures were set up in triplicate and the entire experiment was repeated three times. The average concentration of cells measured every 24 h in all nine independent experiments (*n* = 43,610 total cells analyzed) are shown with box and whiskers plots at each time point. The corresponding *k*-values (finite growth rates) are: AX4 WT shaking 0.034, KO shaking 0.027, AX4 WT stationary 0.021, KO stationary 0.025. b) Single-cell diameter distribution in WT and KO cells. The stacked histogram shows the diameter of single WT (AX4, blue) and DNMA KO (DnmA-, gray) vegetative cells in the proliferation assay. Kolmogorov–Smirnov (K–S) and Chi-square (χ^2^) goodness of fit tests confirm that the two populations of cells are statistically different. c) Visualization of the nuclei in WT and KO cells. DAPI-stained fluorescence images of wild-type (AX4) and *dnmA* knock-out (DnmA-) vegetative cells in both stationary and shaking cultures reveal nuclear abnormalities in the KO cells, including multinucleate and giant cells (scale bar = 5 μm). d) Single-cell nuclear counts. The bar plot shows the number of DAPI-stained nuclei per cell for WT (AX4) and *dnmA* KO (DnmA-) vegetative cells in both stationary and shaking cultures. Cell staining was set up on six slides and the entire experiment was repeated four times. From each slide approximately 100 cells were randomly selected and counted (*n* = 4,937 total cells analyzed).

To investigate if there is a difference between the proliferation of AX4 WT and *dnmA* KO cells we compared the growth curves for both stationary and shaking cultures of vegetative cells over a period of 6 d. WT cells showed a faster growth rate in both conditions when compared to KO cells (Fig. 2a). The KO cells exhibited a measurable lag in proliferation after 48 h in shaking cultures and this difference persisted over the course of the experiment (Fig. 2a). The difference in proliferation rates was less in stationary cultures, but growth was still found to be slower in the KO cells when compared to WT (Fig. 2a). Additionally, the KO cells demonstrated a noticeable heterogeneity in cell size, with multiple larger single cells observed, when compared to the more homogenous population of WT cells. Quantitative analysis of the single-cell diameter distribution between WT and KO cells over the entire time course of the experiment revealed a statistically significant difference, KS test (*P* < 0.05***) and χ^2^ test (*P* < 0.05*) (Fig. 2b). WT cells demonstrated a standard uniform distribution with an average cell diameter of 11.20 μm (SD = ±3.10 μm, *n* = 24,873), while the average diameter size for KO cells was 12.85 μm (SD = ±4.25 μm, *n* = 18,737). When plotted on a histogram, the WT cells show a symmetrical distribution with a peak at 10 to 11 μm. In comparison, the KO cells exhibit a broader peak with a left-skewed mesokurtic distribution, indicating the presence of relatively large cells (i.e. several microns larger than the 10 to 11 μm average for WT cells) within the population (Fig. 2b). Overall, these results indicate that while the DNMA protein is not essential for cell proliferation, its loss is associated with decreased growth rates and cell morphology defects.

### Loss of DNMA results in cell and nuclear morphology phenotypes

We hypothesized that the slower growth rate and size abnormalities in the KO cells might be caused by issues in cell cycle progression, potentially including impaired cytokinesis. In order to test this hypothesis, we analyzed the number of nuclei in both WT and KO vegetative cells that were stained with DAPI. Examination of fixed cells revealed a prevalence of mono- or bi-nucleate cells in WT cells cultured in either stationary or shaking conditions (Fig. 2c). Approximately 82% to 86% of WT cells displayed either a mono- or binucleated phenotype, with the remaining 14% to 18% showing, in decreasing order of frequency, a tri-, tetra- and pentanucleate phenotype (Fig. 2d). Less than 2% of all WT cells exhibited a hexanucleate phenotype and not a single cell was observed with more than 6 nuclei in WT cells (Fig. 2d). Conversely, in the KO cells we found only 73% featured either a mono- or binucleated phenotype, with the remaining 27% consisting of cells with 3 or more nuclei (Fig. 2c). Approximately 10% of all the KO cells displayed a multinucleate phenotype that consisted of nuclei counts ranging from 6 to 32 (Fig. 2d). The occurrence of the 6+ nuclei phenotype in KO cells was primarily observed in noticeably larger (giant) cells, supporting the earlier cell size observation from the proliferation assay.

In addition to differences in nuclei counts between the WT and KO cells, morphological abnormalities were also detected. In WT cells, the nuclei display a relatively homogenous round appearance with an approximate diameter of 3 μm in all cells (Fig. 2c). In KO multinucleate cells, the nuclei are approximately 5 to 8 μm in diameter and their morphology is heterogeneous, frequently displaying varying sizes within a single cell (Fig. 2c). In addition, the cytoplasm to nuclei ratio in KO multinucleate cells displayed statistically significant differences when compared to WT cells (Supplementary Fig. 2), Mann–Whitney *U* test (*P* = 2.61 × 10^−29^), and the giant KO cells had nuclei scattered throughout the cytoplasm (Fig. 2c). These phenotypic abnormalities were not observed in KO mononucleate cells, which have nuclei diameters and cellular morphology similar to WT. Overall, the loss of DNMA in the KO cells is associated with pleiotropic cellular and nuclear phenotypes when compared to WT cells. The results support the hypothesis that some of the KO cells are increasing in size and becoming multinucleated due to a specific failure to complete normal cytokinesis that usually follows karyokinesis during cell cycle progression.

### Immunofluorescence reveals disrupted nuclear and centrosome organization in DNMA KO cells

**Fig. 3.**
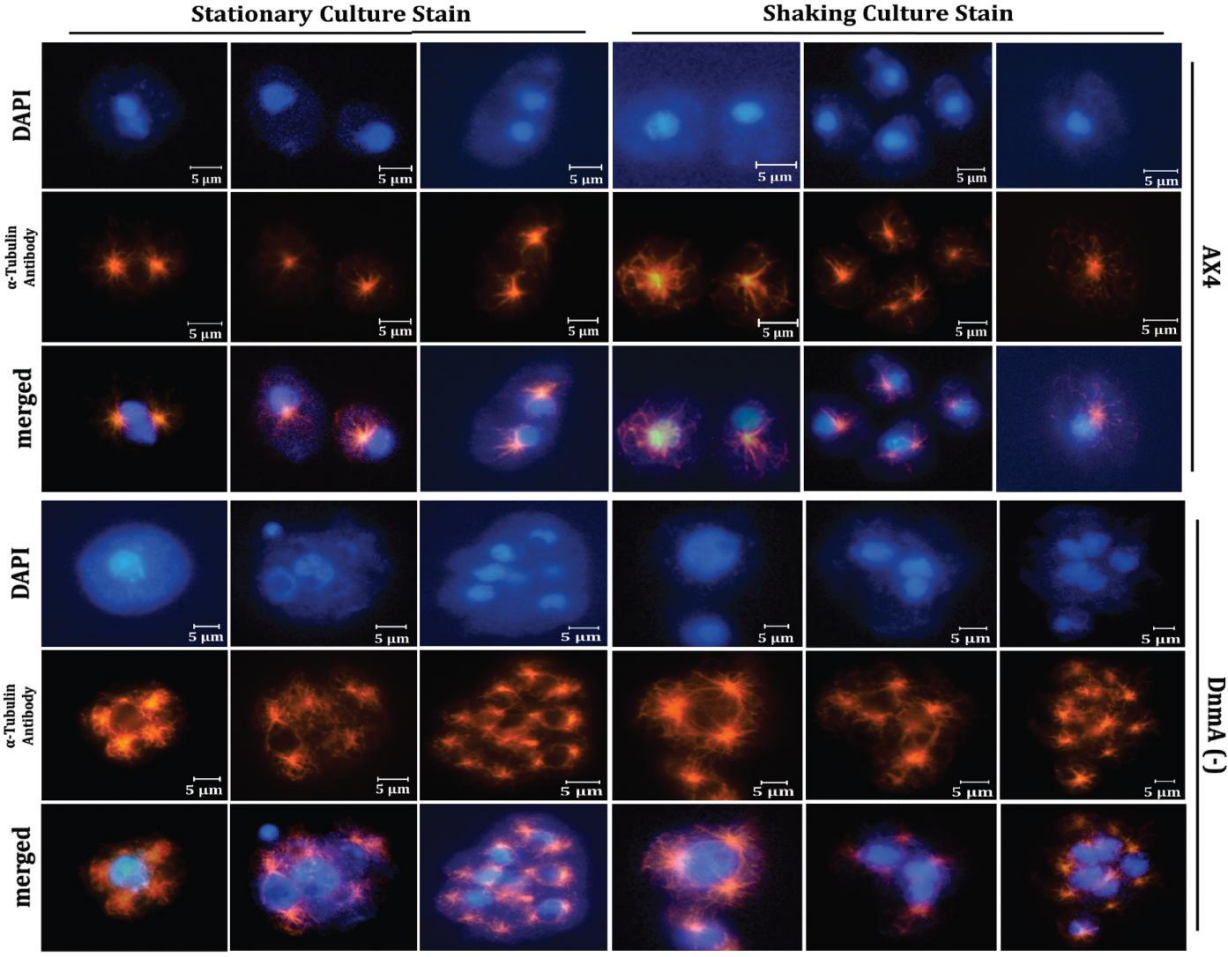
Visualization of centrosomes and spindle bodies in DNMA KO cells. The figure illustrates α-tubulin antibody staining to investigate the effects of *dnmA* KO (DnmA-) on microtubule organization, when compared to WT (AX4) cells. Additional cytosolic microtubules and the prevalence of supernumerary centrosomes at a single nucleus, either in multinucleate or mononucleate cells can be seen the KO cells in contrast to WT cells. The vegetative cells were allowed to develop to confluency in either shaking or stationary conditions prior to fixation and then stained with an antibody to α-tubulin (red) and DAPI (blue).

To further investigate the aberrant nuclear phenotypes in the KO vegetative cells, we compared the microtubule cytoskeleton in WT and KO cells. To do this, we used antiserum against α-tubulin to observe both microtubules and the position of the microtubule-organizing center (MTOC). The alpha-tubulin Ab staining revealed a strong fluorescence peak that was closely attached to the nucleus, corresponding to the MTOC, with attached microtubules (Fig. 3). Upon entry into mitosis, the MTOC is firmly and tightly attached to the nucleus, where it duplicates during prophase and is brought into a fenestration of the nuclear envelope (NE) (Ueda *et al.* 1999). Typically, centrosomes undergo a single duplication event per cell cycle. This observation was confirmed in the WT AX4 cell line, where single nuclei were closely associated with a single MTOC, except in cells undergoing mitosis. However, this observation did not hold for KO cells and a large majority displayed MTOC organization defects. The most common defect observed was supernumerary MTOC, where centrosomes were hyper amplified, yielding varying ratios of at least 1:3 (nuclei: MTOC). The centrosome-to-nucleus ratio was aberrant in both multinucleated (7–32 nuclei) and enlarged (3–5 nuclei) KO cells. In addition, some cells contained supernumerary MTOCs spatially separated from the nucleus and randomly distributed throughout the cytoplasm.

In the KO line, the cytoplasm to nuclei ratio seemed not to be proportional between multinucleate and mononucleate cells and some nuclei in large cells displayed an abnormal morphology characterized by a non-spherical shape and/or fragmented appearance. We initially considered that the aberrant nuclear morphology could be due to NE distortions which have been previously reported to be dynamic (Batsios *et al.* 2019), consistent with the fenestrated mitosis that *D*. *discoideum* cells undergo. Consequently, we examined if the *dnmA* KO affected the overall architecture of the NE using antiserum against DdSUN1. Unlike other SUN1 homologs that are solely localized to the inner nuclear membrane (Lee and Burke 2018), DdSUN1 is an endogenous nuclear membrane protein that is localized to both inner and outer nuclear membranes and remains tightly associated with the centrosome and pericentrosomal region throughout the entire cell cycle (I. Schulz *et al.* 2009). DdSUN1 has an established role in anchoring the centrosome to the nucleus and is a major component of the linker of nucleoskeleton and cytoskeleton (LINC) complex (I. Schulz *et al.* 2009).

WT cells displayed a consistent DdSUN1 localization pattern at the NE, with enhanced localization at the centrosome, generating a staining pattern sometimes referred to as a diamond ring (Supplementary Fig. 3). This position was also visualized using a relative fluorescence unit (RFU) plot, where a sharp peak at the MTOC association site was observed. In WT cells, the DdSUN1 protein localization pattern was essentially identical in mono-, bi-, tri-, tetra-nucleate cells. In contrast, under identical image acquisition settings, KO cells displayed an apparent overexpression and more uniform distribution of DdSUN1 protein in the NE. The pattern was especially notable in the KO cells with aberrant nuclear morphologies as they lacked a distinctive sharp DdSUN1 RFU peak.

To more directly observe any centrosome–NE defects due to the loss of DNMA, we stained cells with antibodies against DdCP224 (a centrosome marker) and DdNE81 (a nuclear lamin-like protein) (Supplementary Fig. 4). WT cells displayed the expected 1:1 ratio of centrosome-to-nucleus, with the centrosome tightly associated with the NE. KO cells, on the other hand, typically contained supernumerary centrosomes, often not associated with any nuclei. Furthermore, the fluorescence intensity of DdCP224 staining was often more diffuse in the KO cells and varied from cell to cell, as well as between centrosomes in cells containing multiple centrosomes. Curiously, in a few KO cells we also observed distention of the NE with a single centrosome at the terminus cells. Whether these nuclear protrusions were formed due to forces acting on the nucleus or the centrosome is not clear and can likely only be identified by live-fluorescence analysis.

The microtubule filaments in *Dictyostelium* are not as dynamic as in mammals, exhibiting growth and shrinkage only at the periphery during interphase (Tikhonenko *et al.* 2016; Koonce 2020). Upon entry into mitosis, centrosomes lose their corona, and microtubules behave dynamically to form central spindles and astral microtubules (Koonce and Tikhonenko 2018). In fluorescence staining experiments with DAPI and α-tubulin, we observed a hyperproliferation of microtubules in KO cells compared to WT cells. With confocal imaging, we were able to reconstruct the filaments to investigate if the dysregulated microtubule network in KO cells was associated with bi-polar spindle formation during *Dictyostelium* mitosis (Supplementary Fig. 5). The reconstruction of Z-stack images indicates that only nucleus-associated centrosomes were associated with the astral microtubules during cell division. In contrast, the cytosolic microtubule network reached the nucleus, but did not appear to impact the nuclear membrane shape or positioning. Interestingly, supernumerary centrosomes located away from any nucleus, likewise displayed hyperpolymerized microtubules. Overall, these experiments present evidence that the loss of DNMA affects nucleus–centrosome–microtubule organization and linkage. Future experiments utilizing live-cell imaging should help to shed further light on these molecular events.

### Loss of DNMA results in developmental defects

**Fig. 4.**
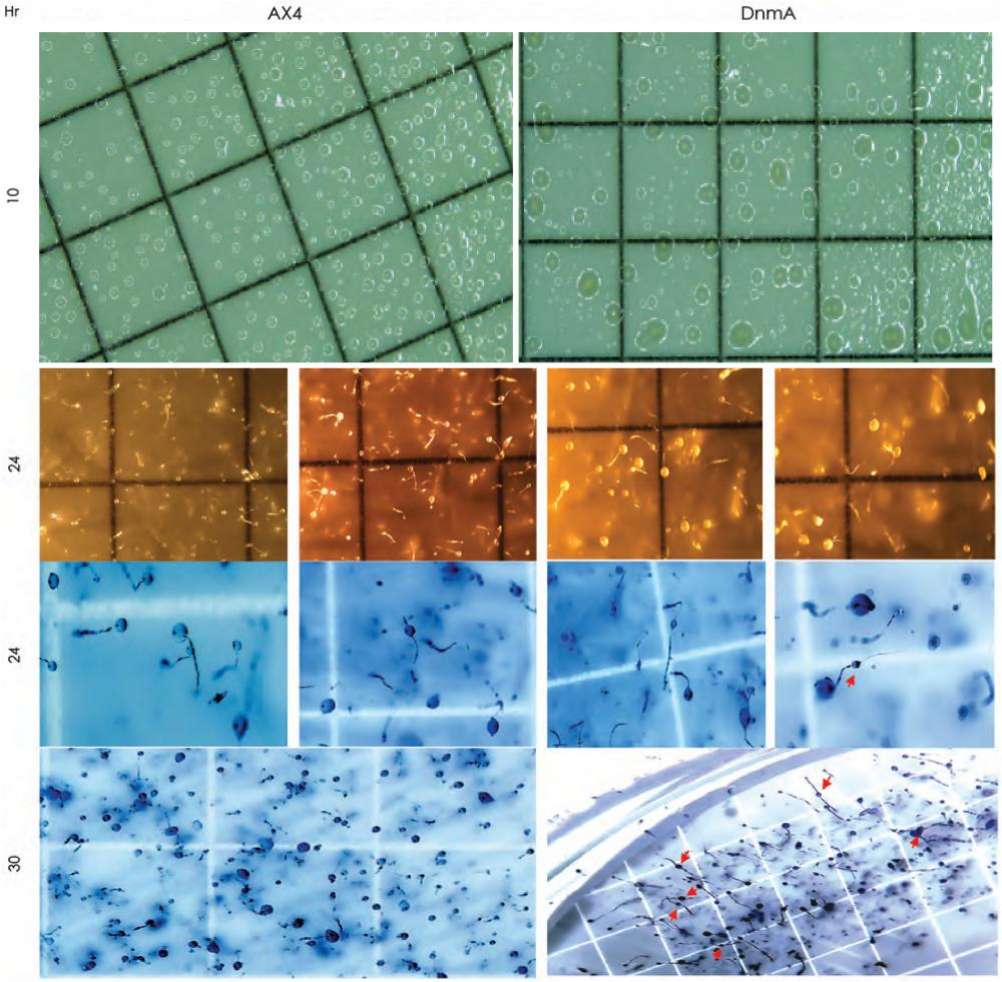
Developmental phenotypes in DNMA KO cells. Comparative analysis of the development of *D. discoideum* WT (AX4) and DNMA KO (DnmA) cell lines at 10, 24 and 30 h post-starvation (membrane grid square = 3.1 mm). Differences in the size and uniformity of the forming aggregates are observed at 10 h and defects associated with the migration of the sori along the stalk in the fruiting body are seen at 24 and 30 h (red arrows). (membrane grid square = 3.1 mm).

*Dictyostelium* is widely studied for its multicellular development program as a response to starvation. Upon nutrient depletion, solitary vegetative cells enter a socially coordinated developmental sequence that is extensively regulated by 3′5′-cyclic adenosine monophosphate (cAMP) (Robertson and Grutsch 1981; Varnum and Soll 1981). We investigated if there are development defects in the *dnmA* KO line when compared to WT. Near confluent cell cultures at 1–3 × 10^7^ cells/ml were plated on gridded membranes and allowed to proceed through the developmental program, with the cells monitored every 6 to 10 h over a 2-d period (see Methods for full details). At 10 h into development (aggregative mound stage), clear morphological differences between the two cell lines were observed. In the WT line, the forming mounds were relatively compact and uniform in size. In comparison, the mounds in the KO line were more irregular in both shape and size (Fig. 4). By 24 h into development the fruiting body is typically fully formed at the end of the culmination process, as was consistently observed in the WT line (Fig. 4). Strikingly, in the KO line we observed many examples of the spore mass migration halted approximately halfway up the stalk. This aberrant phenotype persisted through 30 h and was characterized by a clear separation between the spore mass and the tip of the stalk (Fig. 4, red arrows). The tip is known to control intercellular cAMP signaling during culmination stages (Morimoto 2024).

To further investigate developmental differences, we utilized a live imaging system that enabled us to observe the real-time dynamics during development and measure relative movement in aggregation streams, mounds and slugs (see Methods for full details). In both WT and KO cells, formation of pre-aggregates was seen by 2 to 4 h, followed by mass cell streaming to initially form loosely arranged mounds by 8 h. The mounds proceeded to eventually form a compact hemispherical structure by 18 h and these tight mounds started to form the tip that is thought of as the crucible of cellular differentiation and morphogenesis (Kessin 2001; Dormann *et al.* 2002). Once the tip starts to elongate, increasing amounts of pre-stalk cells partition into the apical region to form a standing structure called the “first finger”. In the WT line, some tight aggregate mounds produced two “first finger” tips, as has been previously reported (Dormann *et al.* 2002). On such occasions, two possible outcomes were observed, either one of the tips dominates while the other eventually disappears, or the two tips orient themselves in opposing directions, continue to grow and this results in subdivision of the mound. In the KO line, the presence of two “first finger” tips was also recorded. However, the KO mounds that produced more than one tip were found not to subdivide successfully. On the contrary, these mounds appeared to attract other migrating pseudo-plasmodia (slugs) and multiple migrating bodies would coalesce, ultimately leading to failure of development as culmination did not proceed properly. In WT lines, culmination proceeded as expected with the conversion of the migratory slug to an upright spore mass containing the spore cells. During the ascension of the pre-spore cells, a typical spiraling movement in the upward clockwise or anti-clockwise orientation was observed in the WT line. However, in the KO line, as the stalk rises it frequently became constricted near the top, which appeared to compromise its ability to support the spore mass. The resulting phenotype may reflect an improper distribution of pre-stalk and pre-spore cells and was characterized by a clear separation between the spore mass and the tip of the stalk, as observed in our earlier studies (Fig. 4, red arrows).

### Quantitative analysis of cell movement during development reveals reduced motility in DNMA KO

**Fig. 5.**
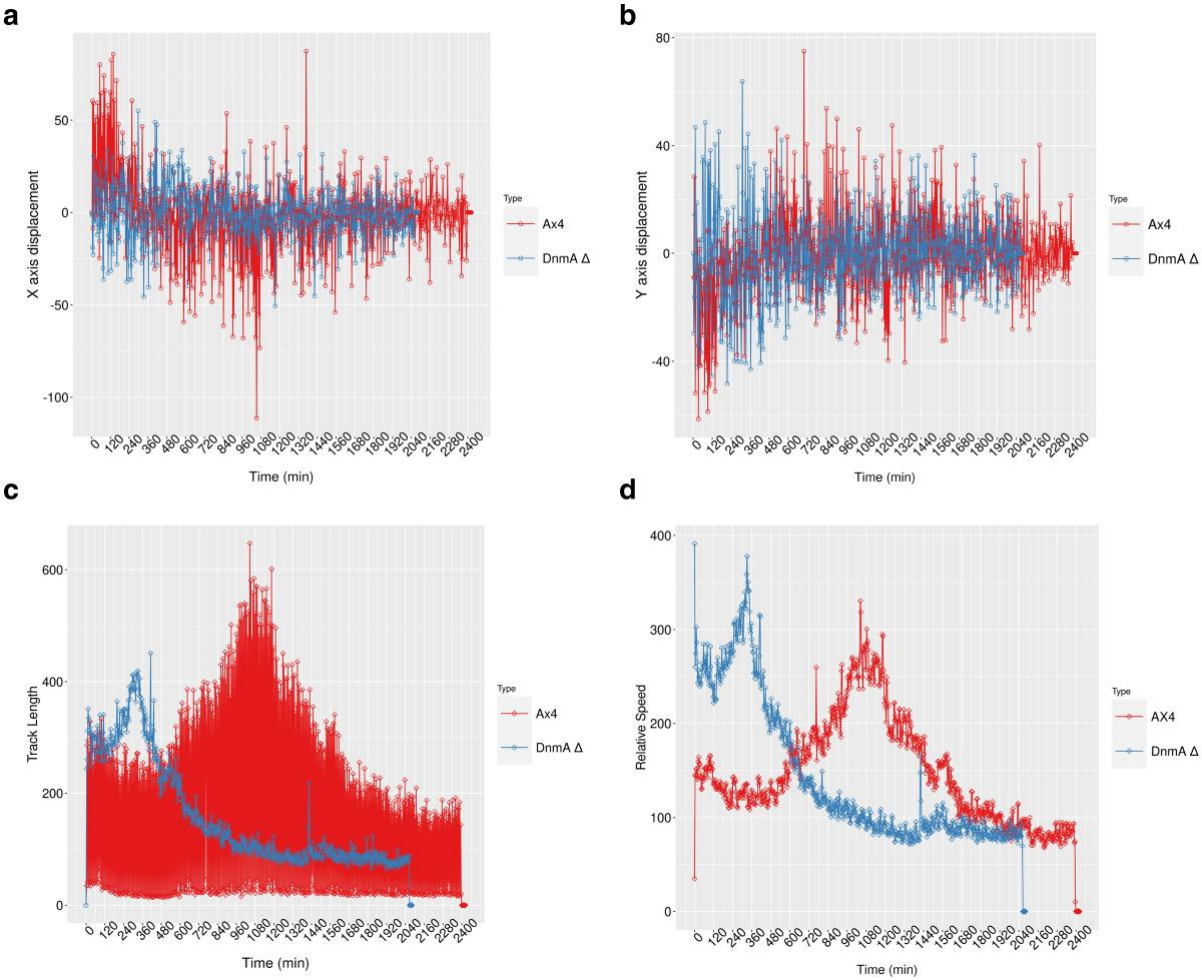
Quantitative comparison of motility during development in WT and DNMA KO cell lines. Pixel spots, corresponding to individual WT (AX4, red) and DNMA KO (DnmA, blue) cells, were identified at the beginning of development and their X-axis (a) and Y-axis (b) displacements tracked over 2,400 min (one pixel = 9.54 μm). c) Relative track length data were extracted from the recorded footage using Imaris Bitplane software revealing that the range is greater in wild-type than knock-out cells and that the timing of peak track length also differed between the two cell types. d) Relative speed data show that both cell types demonstrate a similar range and average peak speed, but that the timing of when the highest relative speeds are detected is distinct between the two cell types, with the KO cells peaking much earlier.

The live imaging footage was processed digitally to allow us to algorithmically reconstruct spot tracks over time (Supplementary Fig. 6). Analysis of two-dimensional (2D) X–Y axis displacement demonstrated no clear differences in the overall directionality of tracked cell movement between WT and KO lines over the developmental time course (Fig. 5, a and b). However, marked differences in cell motility could be observed between WT and KO lines when the track length and relative speed were analyzed. Overall, the WT cells demonstrate a much greater range of track length when compared to KO cells, indicating that the WT cells are in general more motile (Fig. 5c). Furthermore, the peak values for WT track length occur approximately 1,080 min (18 h) into development, which corresponds to the transition from migratory slug to tipped mound stage. In contrast, the peak track length for KO cells occurs at only 240 min (4 h) into development, corresponding to the early aggregation stage (Fig. 5c). After this peak, the relative track length decreases in the KO line, indicating a decreased motility in these cells as development proceeds. The relative speeds displayed by the two different lines mirror the timing of the track length measurements, albeit with a similar maximum relative speed (∼300) displayed by both WT and KO cells (Fig. 5d). This result suggests that the KO cells are not necessarily compromised in their inherent ability to move or the speed with which they move, but rather there is a failure to respond appropriately to the migratory signals that mediate the normal coordinated formation of multicellular structures during development. Overall, these results demonstrate that the DNMA KO cells exhibit an overall reduced motility and distorted timing of migration during development compared to WT cells.

### Transcriptomic analysis identifies differentially expressed genes

To analyze changes in gene expression resulting from the loss of DNMA, RNA sequencing of *D. discoideum* AX4 WT and *dnmA* KO cells was performed. Mapping the sequencing reads to the *D. discoideum* AX4 reference genome achieved an average mapping rate of 92.5%, underscoring the reliability of the sequencing data (Table 1). Gene-level quantification identified a total of 12,000 expressed genes across all samples.

**Table 1.** Percentage of mapped transcriptome reads per replicate for differential gene expression analysis.

Replicate	Wild-type				*DnmA* knock-out			
	WT1	WT2	WT3	WT4	KO1	KO2	KO3	KO4
Read mapping percentage	98.70	98.74	98.76	98.59	98.38	98.51	98.72	98.61

**Fig. 6.**
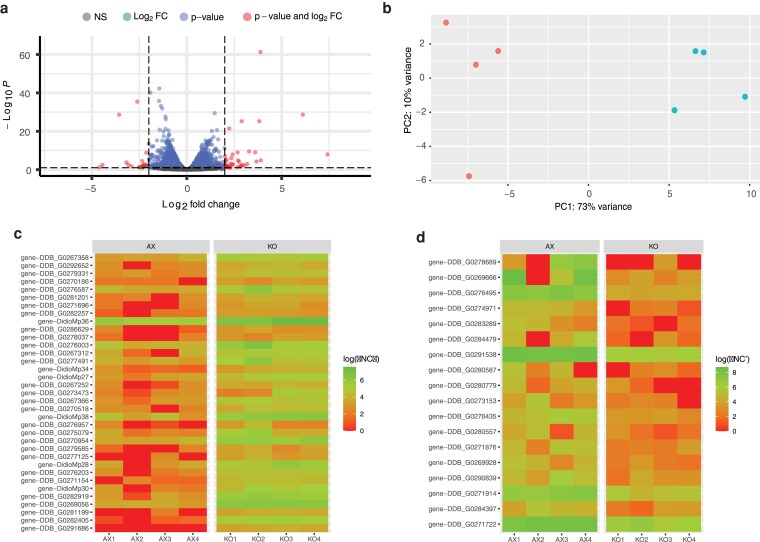
Differential gene expression in WT and DNMA KO cells. a) Volcano plot of gene expression in WT and DNMA KO cells. Most genes (blue circles) do not show significant differences in level of expression between the two cell types. Genes with significant expression changes (adjusted *P*-value < 0.1 and |log₂ fold change| > 2 or < −2) are indicated (red circles). b) Principal Component Analysis (PCA) showing clustering of the four biological replicates each for WT (red) and DNMA KO (blue) cells, with PC1 and PC2 accounting for 73% and 10% of the variance, respectively. c) Heatmap of gene expression in each biological replicate for the 28 genes found to be significantly upregulated in KO (KO1-4) cells when compared to WT (AX1-4) cells. d) Heatmap of gene expression in each biological replicate for the 18 genes found to be significantly downregulated in KO (KO1-4) cells when compared to WT (AX1-4) cells.

Differential expression analysis revealed 46 genes with significant changes in expression between WT and KO samples (Fig. 6a). Of these, 28 genes were upregulated (Table 2), while 18 genes were downregulated in the KO samples compared to the WT (Table 3). PCA demonstrated clear clustering of biological replicates within the WT and KO groups (Fig. 6b), with the first two principal components accounting for 75% of the total variance. This clustering indicates distinct transcriptional profiles between the two groups.

**Table 2.** Differential expression statistics for the 28 significantly upregulated genes from DNMA KO cells compared to WT cells using DESeq2.

Gene	Gene name	Synonyms	Gene products	baseMean	log2FoldChange	lfcSE	stat	*P*_value_	*P*_adj_
DDB_G0291686	DDB_G0291686_RTE	NA	TRE3-B ORF2	17.024	7.414	1.163	6.377	1.81E−10	1.24E−08
DDB_G0282405	DDB_G0282405_RTE	NA	TRE5-A ORF2	94.994	6.114	0.516	11.859	1.94E−32	2.05E−29
DDB_G0281199	DDB_G0281199	NA	unknown	19.308	3.904	0.783	4.987	6.13E−07	1.58E−05
DDB_G0269056	DDB_G0269056_RTE	NA	TRE5-A ORF2	282.924	3.886	0.226	17.159	5.43E−66	5.15E−62
DDB_G0282919	DDB_G0282919_TE	NA	Tdd-4	142.995	3.821	0.343	11.138	8.17E−29	5.54E−26
DDB_G0271154	DDB_G0271154_TE	NA	thugS element	36.661	3.604	0.530	6.796	1.08E−11	1.02E−09
DDB_G0276203	DDB_G0276203	NA	NA	48.190	3.281	0.471	6.961	3.38E−12	3.52E−10
DDB_G0277125	hspG11	NA	heat shock protein Hsp20 domain-containing protein, putative alpha-crystallin-type heat shock protein	15.908	3.027	0.888	3.407	0.000655861	0.005398762
DDB_G0279585	DDB_G0279585	NA	NA	14.711	2.917	0.848	3.441	0.000580381	0.004892001
DDB_G0270954	DDB_G0270954_RTE	NA	Skipper GAG-PRO-POL	179.630	2.896	0.260	11.141	7.92E−29	5.54E−26
DDB_G0275079	hspG1	NA	heat shock protein Hsp20 domain-containing protein, putative alpha-crystallin-type heat shock protein	70.362	2.862	0.865	3.310	0.000934241	0.007197147
DDB_G0276957	DDB_G0276957	NA	NA	13.503	2.815	0.996	2.826	0.004718191	0.02550134
DDB_G0270518	DDB_G0270518	NA	unknown	38.892	2.703	0.541	4.994	5.91E−07	1.53E−05
DDB_G0267366	DDB_G0267366_RTE	NA	Skipper GAG-PRO-POL	64.253	2.680	0.395	6.791	1.11E−11	1.05E−09
DDB_G0273473	DDB_G0273473	NA	unknown	40.070	2.675	1.062	2.519	0.011771322	0.051770906
DDB_G0267252	DDB_G0267252	NA	NA	24.418	2.437	0.663	3.677	0.000235575	0.002355996
DDB_G0277491	hspG12	NA	heat shock protein Hsp20 domain-containing protein, putative alpha-crystallin-type heat shock protein	112.587	2.341	0.422	5.550	2.85E−08	1.13E−06
DDB_G0276003	hspG2	NA	heat shock protein Hsp20 domain-containing protein	213.106	2.301	0.587	3.922	8.77E−05	0.001029709
DDB_G0278037	sahA_ps	NA	pseudogene	10.765	2.273	0.924	2.461	0.013835783	0.05852458
DDB_G0286629	DDB_G0286629	NA	NA	11.100	2.238	0.935	2.395	0.016637176	0.06759565
DDB_G0282257	DDB_G0282257	NA	NA	11.912	2.201	0.768	2.867	0.004146154	0.023093397
DDB_G0271696	prtA	M3L	proteosomal alpha-subunit M3	13.519	2.144	0.863	2.484	0.012993731	0.055878342
DDB_G0281201	DDB_G0281201	NA	unknown	22.479	2.102	0.644	3.265	0.001095386	0.008096811
DDB_G0276587	hspG4	NA	heat shock protein Hsp20 domain-containing protein	120.122	2.092	0.407	5.139	2.77E−07	7.91E−06
DDB_G0270186	DDB_G0270186	NA	Ctr copper transporter family protein	12.949	2.088	0.772	2.705	0.006825279	0.034256334
DDB_G0279331	gtaR	NA	putative GATA-binding transcription factor, GATA zinc finger domain-containing protein 18	42.349	2.038	0.426	4.782	1.74E−06	3.84E−05
DDB_G0292652	DDB_G0292652	NA	NA	26.060	2.031	0.580	3.503	0.000459982	0.004061109
DDB_G0267358	DDB_G0267358_RTE	NA	Skipper GAG-PRO	86.354	2.014	0.337	5.975	2.30E−09	1.25E−07

**baseMean** representing the average normalized expression value across all samples; **log2FoldChange** representing the log_2_-transformed fold change in gene expression between conditions; **lfcSE** representing the standard error of the log_2_ fold change estimate; **stat** representing the Wald statistic for differential expression testing; ***P*_value_** representing the raw *P*-value for the significance of the observed expression difference; and ***P*_adj_** representing the adjusted *P*-value (using the Benjamini–Hochberg method) to control for the false discovery rate.

**Table 3.** Differential expression statistics for the 18 downregulated DEGs from the DNMA KO cells compared to WT cells using DESeq2.

Gene	Gene name	Synonyms	Gene products	baseMean	log2FoldChange	lfcSE	stat	*P*_value_	*P*_adj_
DDB_G0271722	DDB_G0271722	NA	putative extracellular matrix protein	188.780	−2.003	0.243	−8.237	1.77E−16	3.72E−14
DDB_G0284397	DDB_G0284397	NA	NA	19.482	−2.102	0.634	−3.315	0.000916223	0.007075571
DDB_G0271914	DDB_G0271914	NA	CMP/dCMP deaminase, zinc-binding domain-containing protein	95.979	−2.134	0.317	−6.723	1.78E−11	1.59E−09
DDB_G0290839	DDB_G0290839	NA	NA	24.208	−2.166	0.590	−3.675	0.000238125	0.002371501
DDB_G0269928	DDB_G0269928	NA	DUF3430 family protein	15.852	−2.254	0.679	−3.319	0.00090476	0.007011951
DDB_G0271876	DDB_G0271876	NA	NA	18.451	−2.297	0.660	−3.481	0.000500004	0.004333822
DDB_G0280557	DDB_G0280557	NA	putative protein serine/threonine kinase, protein kinase, CMGC group	14.015	−2.301	0.839	−2.742	0.00611136	0.031540468
DDB_G0276435	DDB_G0276435	NA	NA	27.980	−2.346	0.488	−4.807	1.53E−06	3.45E−05
DDB_G0273153	DDB_G0273153	NA	putative glutathione transferase	13.171	−2.383	0.840	−2.838	0.004533611	0.024757483
DDB_G0280779	DDB_G0280779	NA	NA	11.189	−2.452	1.029	−2.383	0.017174678	0.069242533
DDB_G0280587	ubqK_ps2	ubqL	pseudogene	11.970	−2.506	0.979	−2.559	0.010503087	0.047582242
DDB_G0291538	DDB_G0291538	NA	transferase hexapeptide repeat family protein, putative acetyltransferase	204.461	−2.611	0.199	−13.141	1.92E−39	3.64E−36
DDB_G0284479	DDB_G0284479	NA	NA	15.257	−2.919	0.992	−2.944	0.00324159	0.018921238
DDB_G0283289	DDB_G0283289	NA	NA	11.919	−3.076	0.860	−3.579	0.000345215	0.003209901
DDB_G0274971	DDB_G0274971	NA	NA	15.321	−3.186	0.723	−4.406	1.05E−05	0.000178967
DDB_G0276495	DDB_G0276495_RTE	NA	TRE5-A ORF2	97.992	−3.566	0.301	−11.839	2.45E−32	2.32E−29
DDB_G0269666	DDB_G0269666	NA	tRNA-splicing endonuclease subunit Sen15	105.912	−4.446	1.251	−3.553	0.000381095	0.003481206
DDB_G0278689	DDB_G0278689	NA	NA	37.893	−4.604	1.870	−2.462	0.01382364	0.05852458

**baseMean** representing the average normalized expression value across all samples; **log2FoldChange** representing the log_2_-transformed fold change in gene expression between conditions; **lfcSE** representing the standard error of the log_2_ fold change estimate; **stat** representing the Wald statistic for differential expression testing; **P_value_** representing the raw *P*-value for the significance of the observed expression difference; and ***P*_adj_** representing the adjusted *P*-value (using the Benjamini–Hochberg method) to control for the false discovery rate.

Gene annotation analysis highlighted a strong representation of biological processes associated with chromatin organization, retrotransposons, RNA processing, and stress response among the 46 differentially expressed genes (Fig. 6, c and d; Supplementary Fig. 7). Functional annotation revealed upregulation of genes associated with transposable elements (TEs) TRE3-B ORF2 and TRE5-A ORF2 in KO cells (Table 2), suggesting a potential increase in retrotransposon activity. Several heat shock protein genes (*hspG1*, *hspG4*, *hspG11*, and *hspG12*) were also upregulated, indicating an elevated stress-related transcriptional response associated with the loss of DNMA (Fig. 6c; Table 2; Supplementary Fig. 7a). The analysis also identified 18 significantly downregulated genes in the *dnmA* KO samples (Table 3). Key examples include DDB_G0271914, encoding a CMP/dCMP deaminase involved in nucleotide metabolism, and DDB_G0291538, a putative acetyltransferase, suggesting disruptions in acetylation-dependent regulation. Additionally, DDB_G0280587, which encodes for ubiquitin K, and DDB_G0269666, a tRNA-splicing endonuclease subunit (SEN15), were downregulated, indicating potential impacts on ubiquitin signaling and RNA processing pathways. Intriguingly, several ribosomal protein-coding genes were also downregulated (Fig. 6d; Table 3; Supplementary Fig. 7b), suggesting potential alterations in translational efficiency in the KO cells. However, it should be noted that half of all the significantly downregulated genes (9/18) lack functional annotation, highlighting gaps in our knowledge regarding their roles and biological functions.

### Sequence and structural alignment of DNMT2 homologs demonstrates extensive conservation

The DNMT2 protein family is highly conserved across a variety of species (Fig. 1a–c). We analyzed the crystal structures of three DNMT2 enzymes previously deposited in the RCSB Protein Data Bank (PDB) (Berman *et al.* 2000): *Homo sapiens* (hDNMT2, ID: 1g55) (Dong *et al.* 2001), *Spodoptera frugiperda* (SfDNMT2, ID: 4h0n) (S. Li *et al.* 2013) and *Entamoeba histolytica* (EhMETH, ID: 3qv2) (E. C. Schulz *et al.* 2012). All of the enzyme structures were resolved using anomalous X-ray scattering at high resolution (1.80 Å (hDNMT2), 2.71 Å (SfDNMT2), 2.15 Å (EhMETH)) in complex with Ado-Hcy (Supplementary Fig. 8), a product resulting from the canonical methyl donor Ado-Met after methyl group transfer (see Fig. 1e). Therefore, despite their small number, these resolved structures provide a wealth of information for structural comparison, including generating structural models in species that do not currently have their DNMT2 protein structure resolved.

Comparison of the basic physiochemical profiles of the DNMT2s (see Methods for details) revealed that DNMA most closely resembled human DNMT2 (Supplementary Table 1). At the amino acid sequence-level, all the DNMT2 proteins demonstrate a high level of overall conservation and the conserved motifs (I–IX) can be readily identified with motifs I, IV, VI, VIII, IX and X showing the highest levels of conservation (Fig. 1a–c). Given the overall conservation, we therefore matched the related DNMT2 protein crystal structures to each other through partial similarity correspondence alignment using the root mean square deviation (RMSD) of atomic positions, where the average Cα atomic backbone coordinates were calculated after local alignment and rigid body superposition. The average RMSD value was found to be 2.14 Å (generally RMSD values less than 2 Å are considered to be indicative of very similar structural identity), with the overall secondary (Supplementary Table 2) and tertiary structures demonstrating high levels of similarity (Supplementary Fig. 9).

### Biophysical model of DNMA derived from DNMT2 homologs

**Fig. 7.**
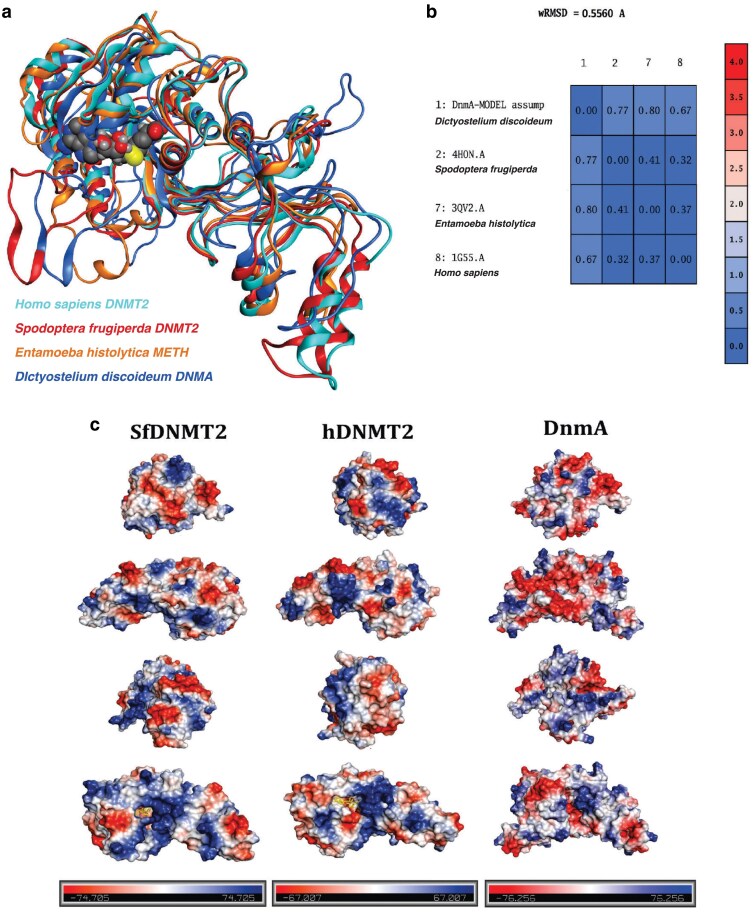
DNMA structure based on sequence alignment and homology modeling. a) Model of DNMA structure. Homology-based model of DNMA (dark blue) superimposed on the experimentally verified X-ray crystal structures of *Homo sapiens* DNMT2 (hDNMT2, ID: 1g55, light blue), *Spodoptera frugiperda* DNMT2 (SfDNMT2, ID: 4h0n, red) and *Entamoeba histolytica* METH (EhMETH, ID: 3qv2, orange) methyltransferases. The DNMA structure was finalized by manually assigning atom coordinates and correcting for loops and deletions/insertions, after which the Boltzmann-weighted assignments and the overall lowest energy conformation was selected. b) Quantitative analysis of structures using weighted root-mean-square deviation (RMSD) of atomic positions. Under standardized environmental conditions, the crystal structures of hDNMT2, SfDNMT2 and EhMETH yielded an average RMSD value of 0.56 Å, with the predicted DNMA structure (DnmA MODEL) closely resembling hDNMT2 (1G55, RMSD = 0.67 Å), SfDNMT2 (4H0N, RMSD = 0.77 Å), and EhMETH (3QV2, RMSD = 0.80 Å). c) The electrostatic potential surfaces of DNMT2 proteins. Electrostatic mapping on the surface of the SfDNMT2, hDNMT2 and DNMA structures is heat color-coded as basic (blue), neutral (white) or acidic (red) according to the assigned electrostatic potential. For surface potentials, the DNMA model was not corrected for loops or deletions/insertions, but was subjected to energy minimization.

Given the overall similarity and high resolution of the DNMT2 X-ray crystal structures and the fact that most structures were resolved within the range of 1.5 to 2.5 Å, indicating that very few residues in the composition have inaccurate rotamers, we reasoned that the existing structures could be used to model the structure of DNMA. Initially, the structures of hDNMT2 and SfDNMT2 were subjected to general Ramachandran structural verification (Supplementary Fig. 10, a and b). Almost all of the residues in both structures were found to be located in the core regions, with only a single outlier residue as measured by ϕ/ψ ratio: hDNMT2 asparagine 325 (Supplementary Fig. 10a). Therefore, both hDNMT2 and SfDNMT2 were selected as templates for template-based modeling of DNMA structure, conducted through comparative homology modeling (as described in detail in the Methods). The relative position of insertions/deletions in the unstructured loop regions located between motif VIII and TR-domain, and between motif X and the CTD (see Fig. 1a–c) were manually adjusted based on the primary amino acid sequence differences between DNMA, hDNMT2 and SfDNMT2. Model inaccuracies were further adjusted to account for distance constraints of the structure, where C-Cα and C-β deviations were corrected through energy minimization. The predicted DNMA model was then subjected to general Ramachandran verification both before and after minimization (Supplementary Fig. 10, c and d). Prior to structural correction, the general ϕ/ψ plot features most residues in the core region at high density. However, a number of residues were located in the allowed region and four residues were detected as outliers (Supplementary Fig. 10c). Manual adjustment and energy minimization improved the predicted structure of DNMA by reducing the outlier number to a single amino acid residue: tyrosine 45 in motif II (Supplementary Fig. 10d). The final predicted DNMA structure was aligned with hDNMT2, SfNMT2 and EhMETH and demonstrated extensive similarity (Fig. 7a). To quantitatively compare the predicted DNMA structure with the experimental DNMT2 crystal structures we analyzed their differences using weighted RMSD. As the X-ray crystal structures were solved at varying temperatures and pH, we standardized to an identical environment (pH = 7.4, T = 310 K). Under these conditions, the crystal structures of hDNMT2, SfNMT2 and EhMETH yielded an average RMSD value of 0.56 Å, with the predicted DNMA structure closely resembling hDNMT2 (RMSD = 0.67 Å), SfDNMT2 (RMSD = 0.77 Å), and EhMETH (RMSD = 0.80 Å) (Fig. 7b).

We mapped the surface potentials on the crystal structures of hDNMT2, SfDNMT2 and the predicted DNMA structure (Fig. 7c). The electrostatic potential had a similar distribution range across all three structures (range: −1.89 to 1.89). Comparison of the electrostatic surface potential distribution in the two experimentally verified structures revealed a basic region around motif X, which is in proximity to the active site of the enzyme. In contrast, this region in DNMA had less basic and more neutral potential distributions (Fig. 7c). The catalytic motifs IV and VI were found to be predominantly neutral in all three structures, while the surface distribution outside of the catalytic regions appears to be relatively evenly distributed (Fig. 7c).

### Docking simulations uncover details of the interaction of DNMT2 with tRNA substrate

The precise molecular details of the interaction between the DNMT2 enzyme and the tRNA^Asp^_GUC_ substrate are unknown, largely because there is currently no co-crystal structure of DNMT2-tRNA in complex available. We therefore utilized *in silico* dynamic simulations and protein docking algorithms to investigate the binding interaction. Based on the secondary structure distribution, the DNMT2 proteins appear to have a large fraction of residues that participate in the formation of unstructured loops or turns (Supplementary Table 2), indicating that these residues could potentially be flexible. Accordingly, we employed an induced fit method in which each backbone movement can affect multiple side chains. The induced fit docking allows the ligand to orient in multi-dimensional space that considers translational, rotational, and conformational variables in the anisotropic environment of the binding site. In addition, the flexible docking algorithms not only often predict the binding mode of a molecule more accurately, when compared to rigid body algorithms, but also output the binding affinity as a function of a change in total entropy (Tessaro and Scapozza 2020). It is, therefore, possible to compare the binding affinities of DNMA to hDNMT2 and SfDNMT2, both of which have previously established experimental evidence to support their binding preference for the tRNA^Asp^_GUC_ substrate (Jeltsch *et al.* 2006, 2017; H. Li *et al.* 2024).

**Fig. 8.**
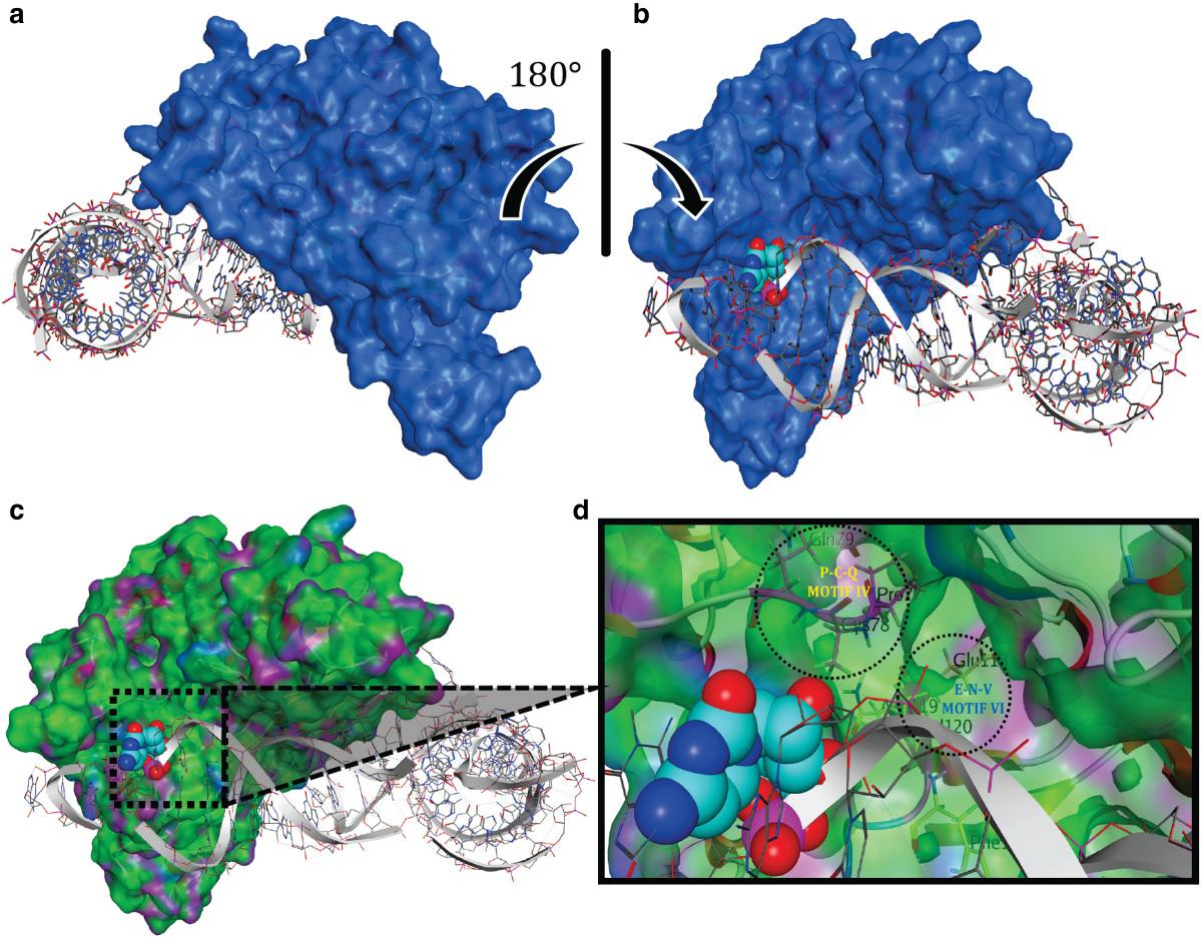
Predicted structure of DNMT2-tRNA complex. a, b) The lowest total potential energy conformation of DNMA bound to tRNA^Asp^_GUC_ in dynamic induced fit docking simulations performed under the potential energy forcefield model of AMBER10: EHT is shown. Dynamic induced fit docking simulation of DNMA-tRNA^Asp^_GUC_ complex (RSEQ = 1, MSEQ = 1, *S* = −75.94 kcal/mol, RMSD = 2.04 Å) with the surface structure of DNMA displayed in blue. The target on the substrate, C38 in tRNA^Asp^_GUC_, is represented as a dummy atom (cyan) in the space-filling model. c) The lowest total potential energy conformation is shown with ActiveLP surface structure coating representing: hydrophobic patches (green), H-bonding with the solvent or neighboring residues (pink), and polar pockets (blue). d) Enzyme-substrate interaction site is shown, with the position of catalytic motif IV (P-C-Q) and motif VI (E-N-V) highlighted (dashed circles). The H-bonding catalytic motifs can be seen in pink under the transparent surface structure.

In general, both protein–ligand binding and protein folding are thermodynamically characterized processes that are primarily driven by the decrease in total Gibbs free energy (ΔG) of the system, where ΔG is the sum of interactions between the ligand and protein corrected for desolvation energies. To examine the dynamic simulations of binding, the enthalpy of the system was set to a constant value, considering access to the solvent, pH, and temperature (see Methods for details). Thus, the only variable in the dynamic simulations was entropy. The binding simulations were conducted on global minimum potential energy conformations of the DNMT2 enzymes and the previously characterized C38 position in the tRNA^Asp^_GUC_ molecule (Jackman and Alfonzo 2013; Torres *et al.* 2014; Jeltsch *et al.* 2017), was identified as the target. In our docking simulations, all three DNMT2s were found to bind to tRNA^Asp^_GUC_ successfully and in strikingly similar structural conformations (Fig. 8; Supplementary Fig. 11). As the hDNMT2 and SfDNMT2 crystal structures contained Ado-Hcy as a co-factor, we incorporated Ado-Hcy in the binding simulations for these two proteins (Supplementary Fig. 11). The predicted structure of DNMA was directly docked to tRNA^Asp^_GUC_ in the absence of Ado-Hcy (Fig. 8). The calculated dissociation constant (K_d_) values for all three DNMT2s suggest relatively weak binding to tRNA^Asp^_GUC_, requiring nearly equimolar concentrations of the binding partners to achieve equilibrium in each case (DNMA *K_d_* = 2.14 × 10^−4^ M, hDNMT2 *K_d_* = 1.89–10^−5^ M, SfDNMT2 *K_d_* = 7.58 × 10^−4^ M). In the predicted bound conformations, all the interactions between DNMT2 and the tRNA were restricted solely to the anti-codon (A) stem-loop and variable arm of the tRNA, indicating that neither the D-loop or T-loop impact the recognition or provide binding stability for DNMT2 (Fig. 8; Supplementary Fig. 11).

## Discussion

### Phenotypes associated with loss of DNMA

Having previously determined that there is little to no measurable DNA methylation in *D. discoideum* (Drewell *et al.* 2023), we set out to characterize the phenotypes associated with the knock-out of the single DNA methyltransferase enzyme (DNMA) identified in the genome of this organism. DNMA is a *bona fide* member of the DNMT2 class of methyltransferases (Jeltsch *et al.* 2017), with an experimentally verified ability to methylate tRNA^Asp^_GUC_ both *in vitro* and *in vivo* (Muller *et al.* 2013). We were interested to see if a more detailed characterization of phenotypes associated with deletion of the *dnmA* gene would not only provide greater insight into the overall biological function of the enzyme, but also identify potential targets susceptible to loss of this enzymatic activity.

Our data do not provide a simple clear-cut definition for a single function of DNMA, but rather show that the *dnmA* KO produces complex pleiotropic phenotypic effects throughout the entire life cycle of *Dictyostelium*, as has been suggested in prior studies (Kuhlmann *et al.* 2005; Katoh *et al.* 2006; Muller *et al.* 2013). This includes a reduced rate of vegetative cell proliferation, especially pronounced in shaking cultures (Fig. 2a). Along with a slower growth rate, we also observed a statistically significant increase in the number of abnormally large cells, suggesting a defect in normal mitotic cell cycle progression and cytokinesis (Fig. 2b). Fluorescence microscopy observations of large, multinucleated KO cells corroborated this finding (Fig. 2c). Indeed, while not a single WT cell was observed with more that 6 nuclei, cells harboring 7–32 nuclei were not uncommon in the KO population (Fig. 2d). The larger KO cells were not only multinucleated, but also sometimes were found to have only a few very large nuclei, typically unequal in size. While earlier studies reached the conclusion that the morphology and temporal proliferation of KO and WT cells were essentially identical prior to culmination (Katoh *et al.* 2006), our results suggest extensive defects in vegetative cells not only in cytokinesis, but in karyokinesis as well. The relatively mild penetrance of disruption of proliferation in stationary vs suspension cultures may suggest more drastic defects in pathways responsible for attachment-independent cell division required for the completion of cytokinesis.

To further explore these defects in cell division we examined additional markers, beyond simply staining DNA to visualize nuclei. In all cases, the KO cells exhibited significant defects when compared with WT cells. These defects include excessive microtubules, supernumerary centrosomes, altered distribution of the NE protein DdSUN1, and distensions of the NE (Supplementary Figs. 3–5). This multitude of phenotypes could function in an additive manner to result in overall defects in cell division. For example, some KO cells were observed to have nuclei attached to multiple centrosomes (Fig. 3) that could create a multi-directional torsional force pulling by the centrosomes that affect nuclear morphology, resulting in nuclear distortions as observed in DAPI and DdNE81 analyses (Supplementary Fig. 5). Multiple centrosomes likely lead to improper segregation of genomic content in subsequent nuclei, as suggested by the nuclear size variations observed in single vegetative KO multinucleate cells.

Further clues as to the molecular nature of the defects are revealed by immunofluorescence staining for DdSUN1. DdSUN1 is normally present in both the inner and outer nuclear membranes and is a key component of the physical connection maintained throughout interphase between centrosomes and centromeres (Batsios *et al.* 2019). The *Dictyostelium* centromeres are telocentric and tightly clustered on the nucleoplasmic side, directly opposite to the centrosome, where they are maintained by a physical linkage that connects the centrosome to the nuclear membrane via the DdSUN1 protein (I. Schulz *et al.* 2009; Leo *et al.* 2012). DdSUN1 staining in our current study indicates that while this protein localizes to the NE successfully in KO cells, the relative distribution of the signal surrounding the envelope is evenly spread (Supplementary Fig. 3). In contrast, in WT cells DdSUN1 staining exhibits distinct clustering at the centrosome/NE interface (Supplementary Fig. 3). Thus, the observed redistribution of the DdSUN1 protein in the KO cells, may be sufficient for aberrant centrosome anchorage or insertion to the NE, which in turn could trigger a delay in proper spindle assembly. In this case, affected cells would exhibit enhanced aneuploidy, possibly due to forces that result from weakened mitotic segregation. This is consistent with our DAPI analysis, revealing abnormally shaped nuclei (Fig. 3). We hypothesize that individual nuclei may be undergoing chromosome duplications at different rates, which may explain the heterogeneous nuclei size within a single cell (Fig. 1c).

The KO cells have been reported to have defects in multicellular development at the culmination stage (Katoh *et al.* 2006). Consequently, our phenotypic analysis was extended to also examine defects in development. Not only did the KO cells take longer to reach culmination, they also had difficulty in producing normally-shaped fruiting bodies, with sorus migration halted approximately halfway up the stalk in many cases (Fig. 4). Furthermore, the analysis of the length and speed of cell migration, from mound to fruiting body, suggests additional developmental defects in the KO cells (Fig. 5). Taken together, our phenotypic analyses are consistent with the idea that the KO cells are compromised in many different biologically impactful pathways, potentially involving a multitude of different genes/proteins, that generate the observable pleiotropic defects.

### Transcriptomic profile

To explore the potential of differential gene expression (either through altered gene transcription or changes in mRNA stability) we turned to RNA sequencing, comparing transcripts from WT and KO cells. Surprisingly, a relatively small number of transcripts differed between the two cell lines (Fig. 6). However, examination of the specific transcripts that were either up- or down-regulated in the KO cells revealed some clear connections to the variety of phenotypes observed in the KO cell line.

Despite the overall low number of genes demonstrating significant transcriptional changes in KO cells, our results reveal prominent upregulation of genes associated with TEs and stress response pathways (Table 2). Among the most highly upregulated genes were TRE3-B ORF2 and TRE5-A ORF2 (Supplementary Fig. 7), which encode retrotransposons, suggesting that the loss of DNMA results in derepression of TE activity. Previous studies show that the depletion of *dnmA* by RNAi leads to upregulation of some retrotransposons (and a reported demethylation at these elements), supporting our results (Kuhlmann *et al.* 2005).

The significant upregulation of heat shock protein genes (*hspG1*, *hspG4*, and *hspG11*) further supports the idea that DNMA loss induces cellular stress, possibly as a response to TE activation or other cellular perturbations. Heat shock proteins play key roles in protecting cells from damage by refolding denatured proteins and maintaining proteostasis under stress conditions, indicating that DNMA deficiency may broadly affect cellular homeostasis (Kuhlmann *et al.* 2005; Miller and Reichow 2025). In addition, as our understanding of tRNA modification evolves from one of a largely static post-transcriptional event to a dynamic regulatory process (Gu *et al.* 2014), the activity of DNMT2 enzymes on their tRNA substrates is now thought to play an important biological role. Indeed, the extent of DNMT2-mediated tRNA modification has been shown to respond to extrinsic stimuli, including stress (Schaefer *et al.* 2010; Endres *et al.* 2015; Mitchener *et al.* 2023; Wadhwa *et al.* 2024), indicating an active role for the molecular regulation of translation as a response to cellular stress.

Beyond TEs and stress response pathways, the transcriptional upregulation of regulatory and transport-associated genes, such as a GATA-binding transcription factor (DDB_G0279331) and a copper transporter (DDB_G0270186), suggests broader impacts of DNMA loss on cellular processes. The GATA-binding transcription factor may drive secondary transcriptional changes that amplify the observed responses (Santhanam *et al.* 2015) while the copper transporter could reflect metabolic adaptations to the altered cellular state (Hatori *et al.* 2016). Interestingly, many differentially expressed genes, including DDB_G0281199 and DDB_G0273473, lack functional annotation, highlighting gaps in our understanding of the *D. discoideum* genome. These uncharacterized genes could represent novel players in pathways affected by DNMA and warrant further investigation.

Taken together, these findings suggest that DNMA has a multifaceted role in regulating genome stability, transposon repression, and cellular stress responses. The insights gained from these results provide a foundation for understanding how DNMA contributes to the maintenance of cellular homeostasis and genome integrity, with potential implications for similar regulatory mechanisms in other organisms. Future work could focus on the molecular pathways by which DNMA exerts its effects and explore its interactions with other chromatin and genome-regulatory proteins to gain a more comprehensive understanding of its functions.

### Homolog-based structural model of DNMA

As there is currently no published crystal structure of DNMA, we developed a structural model of the protein based on homolog to three other well-studied DNMT2s. The primary amino acid sequence, organization of catalytic motifs (Fig. 1) (Liu and Santi 2000; Jeltsch 2002), and physiochemical properties (Supplementary Table 1) share extensive similarity between all four proteins. Furthermore, the secondary structure composition (Supplementary Table 2) and crystal structures (Supplementary Fig. 9) of the experimentally verified DNMT2s are also largely shared. Perhaps not surprisingly, the predicted model structure we were able to derive for DNMA very closely resembles that of the other DNMT2 homologs (Fig. 7), strongly suggesting a high level of evolutionary and functional conservation amongst this group, as has been previously reported (Jeltsch *et al.* 2017).

Assessment of the accuracy of the homology–based model was determined by calculating RMSD values as an average measure of distance between two atoms. The predicted DNMA structure was found to most closely resemble that of hDNMT2 (RMSD = 0.67 Å) at very high resolution, followed closely by SfDNMT2 (RMSD = 0.77 Å) and EhMETH (RMSD = 0.80 Å). Despite the overall sequence and structural similarity, there were some unique features revealed in DNMA when compared to the three other DNMT2s. The C_TD_ of DNMA contains an insertion of low complexity sequence at residue positions 362–379, representing a species-specific variation that is not present in other DNMT2 enzyme homologs (Fig. 1c). Additionally, DNMA does not contain the DNMT2-specific conserved CFT motif within the TR-domain (Cheng 1995; Jeltsch 2002), as the motif is disrupted by the replacement of phenylalanine (F) with valine (V) at residue position 274 (Fig. 1c). Nonetheless, as both valine and phenylalanine residues are hydrophobic, with pI values of 5.96 and 5.48 respectively, they exhibit a similar non-polar electrostatic profile and therefore this amino acid change would most likely not significantly impact any role in target recognition by DNMA. Previous studies have suggested there is some plasticity in this motif, as exchange of C292 in the CFT motif of hDNMT2 reduced, but did not completely disrupt, the catalytic activity of the enzyme (Jurkowski *et al.* 2008).

Electrostatic potential plays a significant role in the activity of any enzyme. Obtaining the electrostatic surface profile of DNMA allowed us to compare it to other DNMT2s (Fig. 7c). Previous studies have reported a lower prevalence of positive potential (basic) in the catalytic domain appears to be a feature of tRNA methyltransferases (Dong *et al.* 2001; S. Li *et al.* 2013; Jeltsch *et al.* 2017). DNMA displays a positive potential close to the active site that is concentrated in a small patch of residues, whereas SfDNMT2 and hDNMT2 are more similar to each other in that they have larger surface potential distributions. In particular, the posterior surfaces of both SfDNMT2 and hDNMT2 exhibit high variance in positive and negative potential, while DNMA displays a large concentration of acidic residues posterior to the active site (Fig. 7c). This 3D electrostatic potential mapping explains why the aliphatic index of DNMA is lower when compared to the other DNMT2s (Supplementary Table 1), and the prevalence of a focused negative potential on the posterior side of the enzyme may be indicative that this enzyme has yet uncharacterized protein binding partners.

### DNMA-tRNA docking simulations

Given the high fidelity of our DNMA structural model, and since there is no DNMT2-tRNA co-crystal structure available, we ran simulations to model the interaction between these two molecules. Previous studies have successfully mapped the tRNA binding site on the surface of hDNMT2 and identified eight basic residues that contribute strongly to catalytic activity (R95, R84, R275, K367, R371, R289, K122, and K295, listed in order of increasing residual activity), all of which cluster in the wall of a cleft on the surface of enzyme (Jurkowski *et al.* 2012; Jeltsch *et al.* 2017). Manual placing of tRNA into the structure suggests that DNMT2 mainly interacts with the anti-codon loop (ACL) (Jurkowski *et al.* 2012). Our models show that all three DNMT2s (DNMA, hDNMT2 and SfDNMT2) are capable of binding tRNA^Asp^_GUC_ in very similar structural conformations (Fig. 8; Supplementary Fig. 11). The previously described binding cleft (Jurkowski *et al.* 2012) is confirmed as the conserved binding pocket in all three enzymes and the interaction is focused on the ACL region of the tRNA molecule. The biophysical arrangement places the target cytosine at position 38 in the tRNA (C38) adjacent to the catalytic domain of the enzyme. In particular, catalytic motif IV (P-C-Q) and motif VI (E-N-V) in the protein are in very close proximity to C38 and form multiple hydrogen bonds with the tRNA substrate. Intriguingly, the calculated dissociation constant (K_d_) values for all three DNMT2s demonstrate relatively weak binding to tRNA^Asp^_GUC_, requiring nearly equimolar concentrations of the binding partners to achieve equilibrium in each case. This result may be indicative of the fact that it is biologically advantageous to have weak binding to facilitate rapid association and dissociation of the enzyme from the tRNA substrate, and/or the binding affinity may be further impacted by, as yet uncharacterized, protein binding partners, similar to other tRNA recognizing enzymes (J. Li *et al.* 2023; Throll *et al.* 2024).

Our binding simulations strongly indicate that the structural arrangement of the molecular interaction between DNMT2 homologs and tRNA is evolutionarily conserved. Given that DNMT2-mediated methylation has been shown to be largely specific for the C38 residue (Goll *et al.* 2006) and that position is close to the wobble base in tRNA (Agris *et al.* 2007), our results support a role for DNMT2s in the regulation of protein translation. Prior studies have indicated that DNMT2-mediated methylation of C38 in tRNA^Asp^_GUC_ provides a mechanism to fine tune translational efficiency for groups of proteins with poly-Aspartic acid (Asp) sequences (Tuorto *et al.* 2012; Shanmugam *et al.* 2015). However, we can not exclude the possibility that DNMA-mediated methylation may not be exclusive to tRNA^Asp^_GUC_, as similar ACL nucleotide sequences are also found in tRNA^val^_TAC_, tRNA^Val^_AAC_, tRNA^Val^_CAC_, and tRNA^Gly^_GCC_, specifically the 5′-C^36^AC^38^GCA/G-3′ motif is present in all of these tRNA species. This leaves open a potential role for additional, as yet unidentified, adapter proteins in modulating the binding specificity of DNMA (Muller *et al.* 2013), perhaps through the selective recognition of sequences in the D- and/or T-loop of the tRNAs. The detailed investigation of this tRNA methylation regulatory activity for DNMT2 homologs, including DNMA in *Dictyostelium*, and the biological connection to the pleiotropic phenotypes associated with the loss of DNMA, should be a high priority for future in this field.

## Supplementary Material

jkaf152_Supplementary_Data

## Data Availability

The sequencing datasets supporting the results of this study are available at the NCBI Sequence Read Archive (SRA) under BioProject accession number PRJNA1193615. The raw data used in the cell phenotype and protein modeling studies in this article are available at the following GitHub site: https://github.com/zazadovv/Dissecting-the-function-of-the-DNMT2-homolog-DNMA-in-Dictyostelium-discoideum.git. Supplemental material available at *G3* online.
