# Supplementary material for: Dissecting the function of the DNMT2-homolog (DNMA) in Dictyostelium discoideum
Source: G3 (Bethesda). 2025 Jul 4;15(9):jkaf152. doi: 10.1093/g3journal/jkaf152 (PMC12405889; doi:10.1093/g3journal/jkaf152)
Supplement: jkaf152_Supplementary_Data [file jkaf152_supplementary_data.zip › Supplemental_Material_Legends_G3-2025-406015.docx]

**Supporting Information**

**Figure S1**

**Confirmation of *dnmA* knock-out by read depth analysis.** Whole genome read depth across the *dnmA* locus (Chr. 5: 1026957–1028573) in WT and KO strains. In the KO strain, median coverage across *dnmA* was 1×, compared to 117× across entire chromosome 5, indicating successful deletion. In WT, coverage was consistent across *dnmA* and chromosome 5 (83× and 97×, respectively). The *trmt5* gene (Chr. 3: 2486411–2488090) served as a control, showing comparable coverage in both WT and KO strains, confirming the specificity of the *dnmA* deletion.

**Figure S2**

**Quantitative analysis of nucleus / cytoplasm area ratio. (A)** Histogram showing distribution of nucleus / cytoplasm area ratio in WT (AX4, blue) and KO (DnmA, orange) cells. **(B)** Boxplots showing nucleus / cytoplasm area ratio in individual WT (AX4, blue) and KO (DnmA, orange) cells.

**Figure S3**

**Visualization of LINC complex component DdSUN1 in WT and KO cells.** The figure illustrates DdSUN1 antibody staining to investigate the effects of *dnmA* KO (DnmA-) on nuclear envelope organization, when compared to WT (AX4) cells. The data was pooled from two independent replicates. Log-phase cells of either WT or KO were fixed and stained as indicated (DAPI, blue and DdSUN1, red). The RFU plots clearly demonstrate that the WT cells always maintain the characteristic unequal ‘diamond ring’ DdSUN1 distribution in the nuclear envelope, even in multinucleated cells. On the contrary, a much more homogeneous localization of DdSUN1 is observed in the KO cell population.

**Figure S4**

**Analysis of DdCP224 and DdNE81 localization in WT and KO cells.** The figure illustrates maximum intensity projection Z-stack images of DdCP224 (green) and DdNE81 (red) localization. Twenty images per stack were obtained. Individual scale-bars are presented in each of the merged images. Both stationary and shaking cultures of WT cells (AX4) display the canonical one centrosome to one nucleus ratio, whereas loss of DNMA in KO cells (DnmA) leads to supernumerary centrosomes.

**Figure S5**

**Analysis α-Tub and DdNE81 in WT and KO cells.** The figure illustrates maximum intensity projections of Z-stack images, revealing the localization of microtubules (green) and DdNE81 (red). The data was pooled from three independent replicates and a total of 25 images per stack were obtained. Individual scale-bars are presented in each merged image. The immunofluorescence signal suggests that loss of DNMA in KO cells (DnmA) leads to higher levels of α-tubulin staining at all stages of the cell cycle when compared WT cells (AX4).

**Figure S6**

**Two-dimensional developmental cell tracing in *Dictyostelium discoideum.*** Cell-based tracing of *D.discoideum* WT (AX4) and *dnmA* KO (DnmA) live cells over 48 hours of development. Images of the cells at the end of the 48 hour period are shown under normal light (Raw Final Snapshot) and inverted (Inverted Colors) for clarity (membrane grid square = 3.1mm). Cell movements were viewed as dragon tail-tracks over time (refer to Methods for detailed description). The tracing algorithm is encoded with a time-related color code; movements detected early in development are displayed in blue-purple, movements in the middle of the recording period are displayed in green, and movements later in development are displayed in yellow-red. This enables the differences in spatial and temporal movements to be traced during development in the two cell types. Red arrows indicate examples of the aggregating cell cluster collapse in the KO cells.

**Figure S7**

**Normalized gene expression profile for all differentially expressed genes. (A)** Boxplots showing normalized expression levels of the 28 genes significantly upregulated in KO (KO, light gray) cells when compared to WT (AX, dark gray) cells. **(B)** Boxplots showing normalized expression levels of the 18 genes significantly downregulated in KO (KO, light gray) cells when compared to WT (AX, dark gray) cells. Each plot is labeled with the annotated gene name and highlights the transcriptional shifts associated with the loss of DNMA.

**Figure S8**

**The binding of Ado-Hcy in the DNMT2 ligand pocket**. **(A)** Visualization of the three-dimensional crystal structure of human DNMT2 (cyan) interacting with the Ado-Hcy ligand (yellow). **(B)**  Visualization of the three-dimensional crystal structure of S. *frugiperda* DNMT2 (red) interacting with the Ado-Hcy ligand (yellow). In both panels the amino acid residues in DNMT2 that are directly interacting with the ligand are labeled (green) and the bonds shown (dashed yellow lines).

**Table S1**

**Predicted physiochemical properties of DNMT2 in different species**. The basic physiochemical profiles of *Dictyostelium discoideum* DNMA (DNMA), *Homo sapiens* DNMT2 (hDNMT2), *Spodoptera frugiperda* DNMT2 (SfDNMT2), and *Entamoeba histolytica* METH (EhMETH) are shown.

**Table S2**

**Secondary structures in DNMT2 crystal structures.** The distribution of different secondary structures in single polypeptide chains from the X-ray crystal structures of three DNMT2 enzymes previously deposited in the RCSB Protein Data Bank (PDB); *Homo sapiens* (hDNMT2, ID: 1g55), *Spodoptera frugiperda* (SfDNMT2, ID: 4h0n) and *Entamoeba histolytica* (EhMETH, ID: 3qv2)*.*

**Figure S9**

**DNMT2 surface structure superposition**. **(A)** Surface structure superposition of DNMT2 proteins from human (PDB: 1g55, cyan), *S. frugiperda* (PDB: 4h0n, red) and *E. histolytica* (PDB:3qv2, orange). The proteins are displayed as both ribbon and surface wire-mesh structures. Although the proteins demonstrate extensive similarity to each other (RMSD = 2.14 Å), several of the differences between the structures are highlighted (dotted circles). The corresponding RMSD matrix values of the positions of the Cα atoms for each pair of proteins, measured after optimal rigid body superposition, are shown. **(B)** Solid superimposed surface structure of hDNMT2 (cyan) and SfDNMT2 (red) confirm the overall structural similarity.

**Figure S10**

**Ramachandran plots for structural validation of predicted DNMA structure**. φ/ψ (Ramachandran) plots for hDNMT2, PDB: 1g55 **(A)** and SfDNMT2, PDB: 4h0n **(B)**, with residues in the core (green circles) and allowed (orange circles) regions indicated. No outlier residues are detected in SfDNTM2 and only a single outlier residue, ASN_325 (red cross), is detected in hDNMT2. The φ/ ψ ratio for this outlier residue is on the boundary between allowed and outlier regions and therefore is unlikely to cause inaccurate folds in the structure. **(C)** φ/ψ (Ramachandran) plot for the template-based predicted 3D structure of DNMA, with residues in the core (green circles) and allowed (orange circles) regions indicated. The four outlier residues are indicated with a red cross. **(D)** φ/ψ (Ramachandran) plot for the predicted 3D structure of DNMA after the backbone segment of the protein was subjected to a series of energy minimizations to relieve steric hindrance and strains under the default forcefield *AMBER10: EHT* (see Methods for details). Residues in the core (green circles) and allowed (orange circles) regions are shown. Energy minimization results in only a single outlier residue, TYR_45 (red cross), detected in DNMA.

**Figure S11**

**Structure of DNMT2-tRNA** **complexes.** The lowest total potential energy conformation of hDNMT2 (PDB: 1g55, cyan) **(A)** and SfDNMT2 (PDB: 4h0n, red) **(B)** bound to tRNA^Asp^_GUC_ in dynamic induced fit docking simulations performed under the potential energy forcefield model of AMBER10: EHT are shown. The Ado-Hcy methyl donor (yellow) is highlighted and the target on the substrate, C38 in tRNA^Asp^_GUC_, is represented as a dummy atom (red for hDNMT2 and cyan for SfDNMT2) in the space-filling models. The two structures demonstrate similar binding profiles; hDNMT2 (RSEQ=1, MSEQ=1, S=-96.98 kcal/mol, RMSD=0.78 Å), SfDNMT2 (RSEQ=1, MSEQ=1, S=-71.88 kcal/mol, RMSD=1.01 Å).
