# Supplementary material for: Dissecting the function of the DNMT2-homolog (DNMA) in Dictyostelium discoideum
Source: G3 (Bethesda). 2025 Jul 4;15(9):jkaf152. doi: 10.1093/g3journal/jkaf152 (PMC12405889; doi:10.1093/g3journal/jkaf152)
Supplement: jkaf152_Supplementary_Data [file jkaf152_supplementary_data.zip › Table_S1_G3-2025-406015.pdf]

Protein characteristic	DnmA	hDnmt2	SfDnmt2	EhMeth2
**Amino acids**	379	391	333	332
**Formula**	$C_{1968}H_{3047}N_{523}O_{608}S_{11}$	$C_{2016}H_{3203}N_{521}O_{588}S_{14}$	$C_{1729}H_{2711}N_{453}O_{495}S_{20}$	$C_{1693}H_{2645}N_{433}O_{493}S_{15}$
**Molecular weight (kDa)**	44.1	44.6	38.4	37.4
**Isoelectric point (pI)**	5.81	5.77	8.35	8.26
**Extinction coefficient (Liter/(Mol·cm))**	0.665	0.501	1.012	0.824
**Instability index (II)**	46.12	51.42	43.26	44.44
**Aliphatic index**	74.56	101.43	88.35	90.78
