# Supplementary material for: Dissecting the function of the DNMT2-homolog (DNMA) in Dictyostelium discoideum
Source: G3 (Bethesda). 2025 Jul 4;15(9):jkaf152. doi: 10.1093/g3journal/jkaf152 (PMC12405889; doi:10.1093/g3journal/jkaf152)
Supplement: jkaf152_Supplementary_Data [file jkaf152_supplementary_data.zip › Table_S2_G3-2025-406015.pdf]

Secondary structure	hDNMT2 (PDB ID: 1g55)		SfDNMT2 (PDB ID: 4h0n)		EhMETH (PDB ID: 3qv2)	
	Residues	Percentage	Residues	Percentage	Residues	Percentage
**α-helix**	101	29.45%	94	26.47%	83	25.38%
**Parallel β-strand**	30	8.75%	27	7.61%	26	7.95%
**Antiparallel β-strand**	21	6.12%	22	6.20%	4	0.90%
**3_{10}-helix**	20	5.83%	18	5.07%	29	8.87%
**π-helix**	0	0.00%	0	0.00%	0	0.00%
**Turns**	38	11.07%	50	14.08%	42	12.84%
**Unstructured**	133	38.78%	144	40.57%	131	40.06%
