# Supplementary figures and images for: Dissecting the function of the DNMT2-homolog (DNMA) in Dictyostelium discoideum
Source: G3 (Bethesda). 2025 Jul 4;15(9):jkaf152. doi: 10.1093/g3journal/jkaf152 (PMC12405889; doi:10.1093/g3journal/jkaf152)

**A**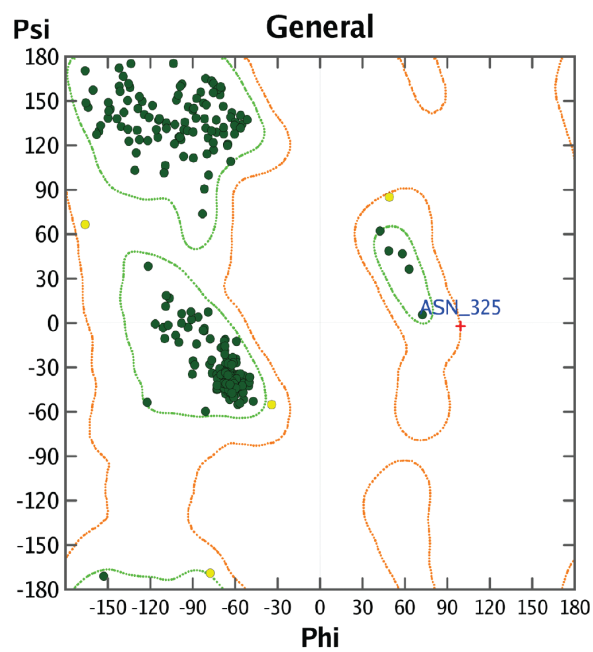**B**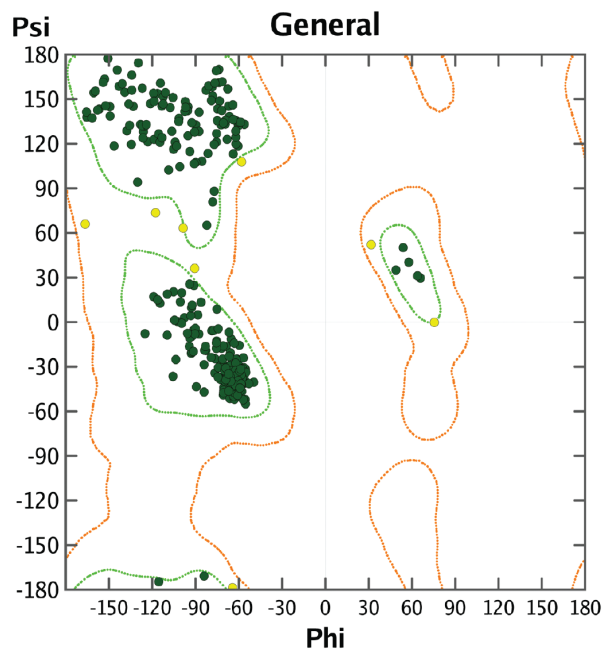**C**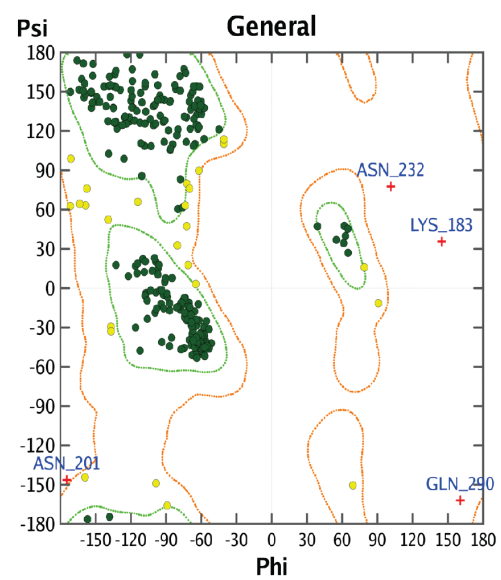

Energy  
Minimization

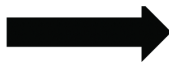**D**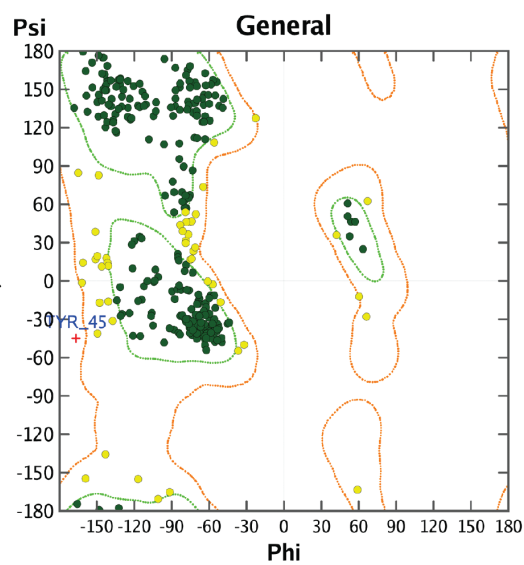

Supplement: jkaf152_Supplementary_Data [file jkaf152_supplementary_data.zip › Figure_S10_G3-2025-406015.pdf]

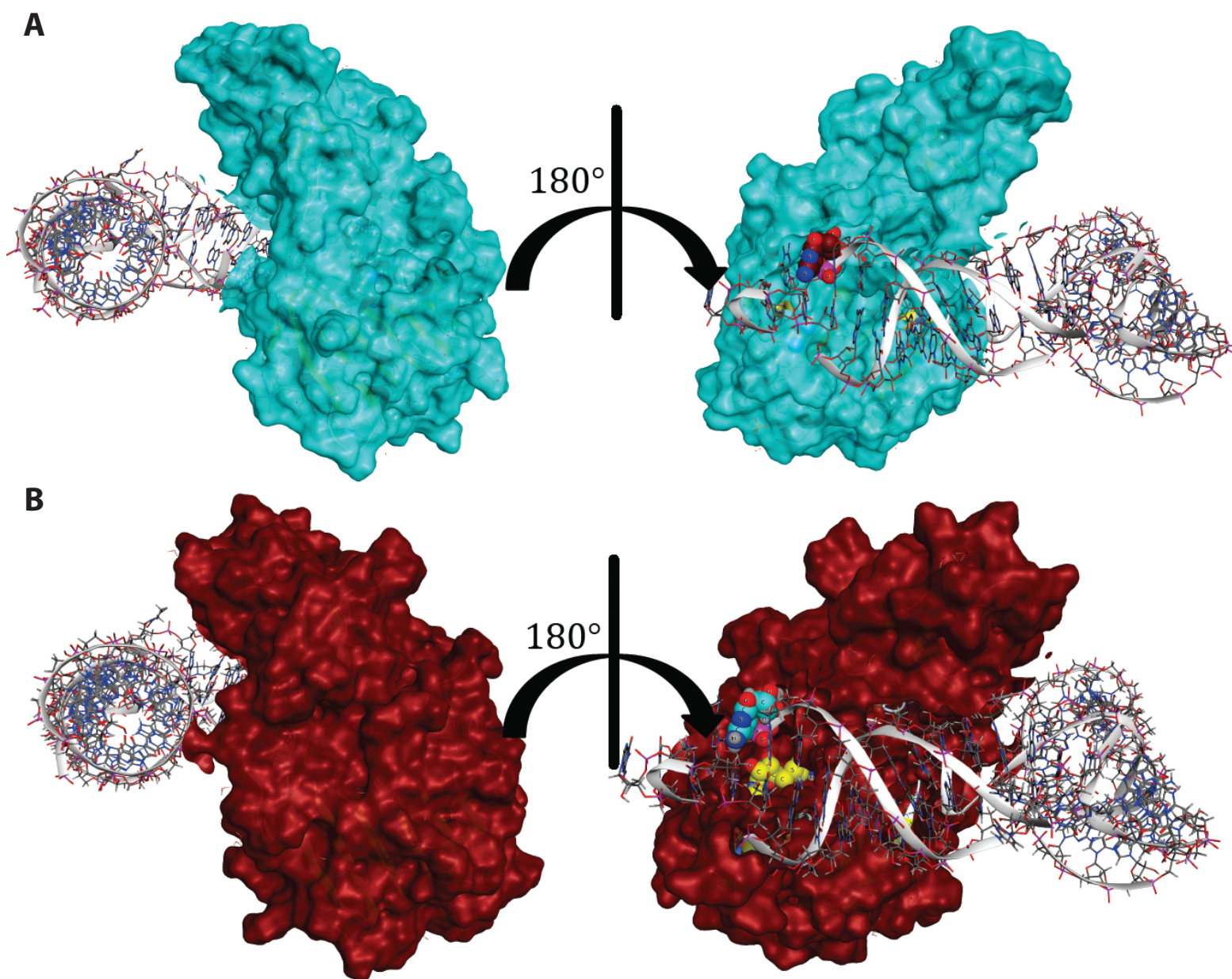

Supplement: jkaf152_Supplementary_Data [file jkaf152_supplementary_data.zip › Figure_S11_G3-2025-406015.pdf]

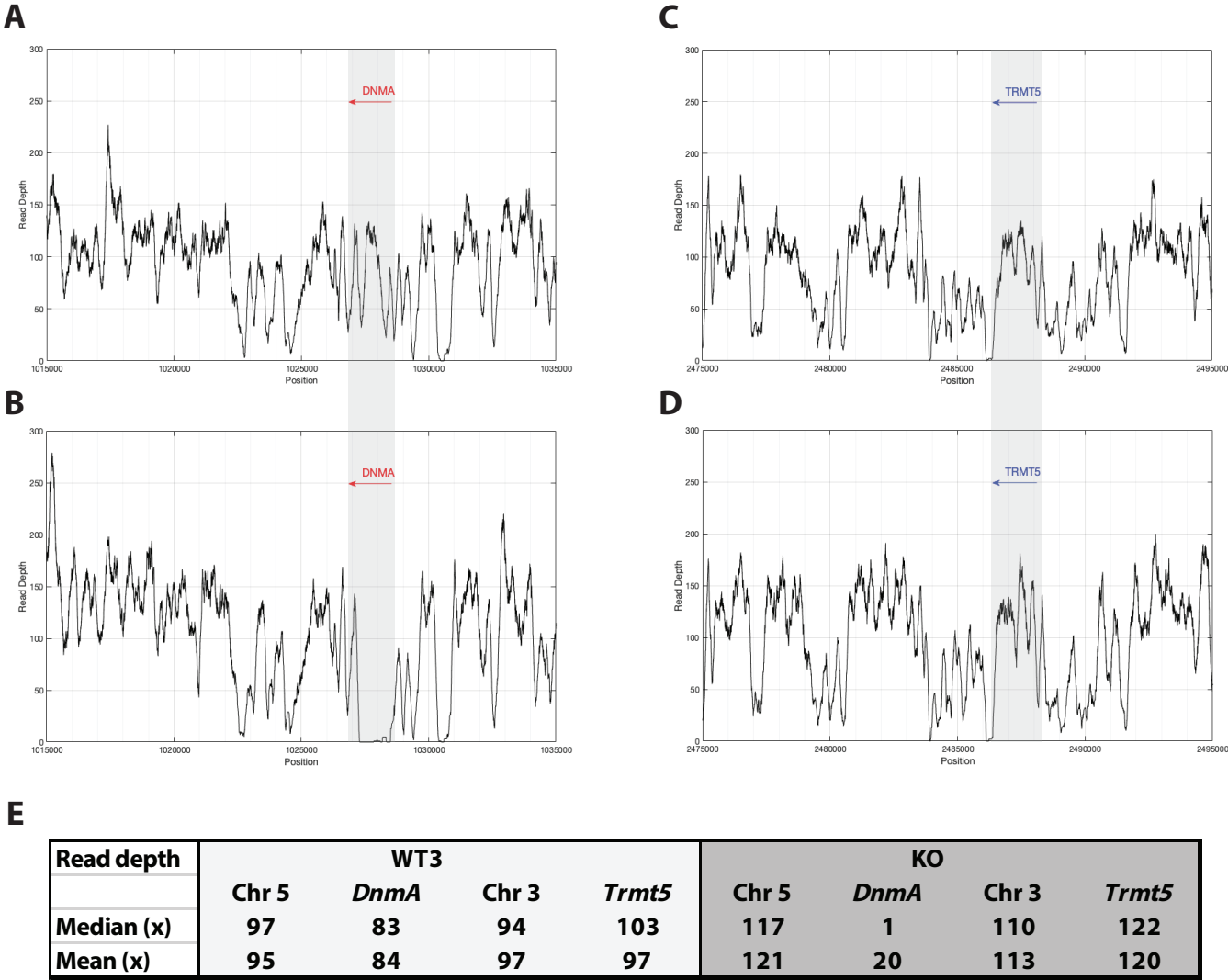

Supplement: jkaf152_Supplementary_Data [file jkaf152_supplementary_data.zip › Figure_S1_G3-2025-406015.pdf]

**A**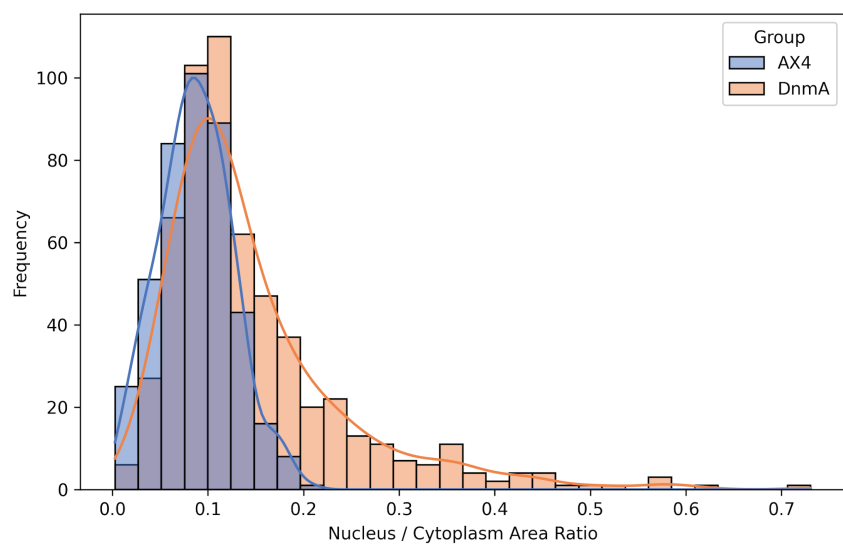**B**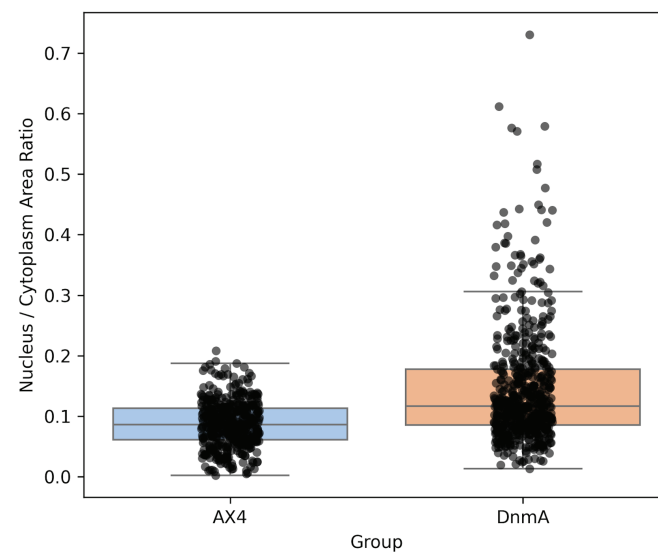

Supplement: jkaf152_Supplementary_Data [file jkaf152_supplementary_data.zip › Figure_S2_G3-2025-406015.pdf]

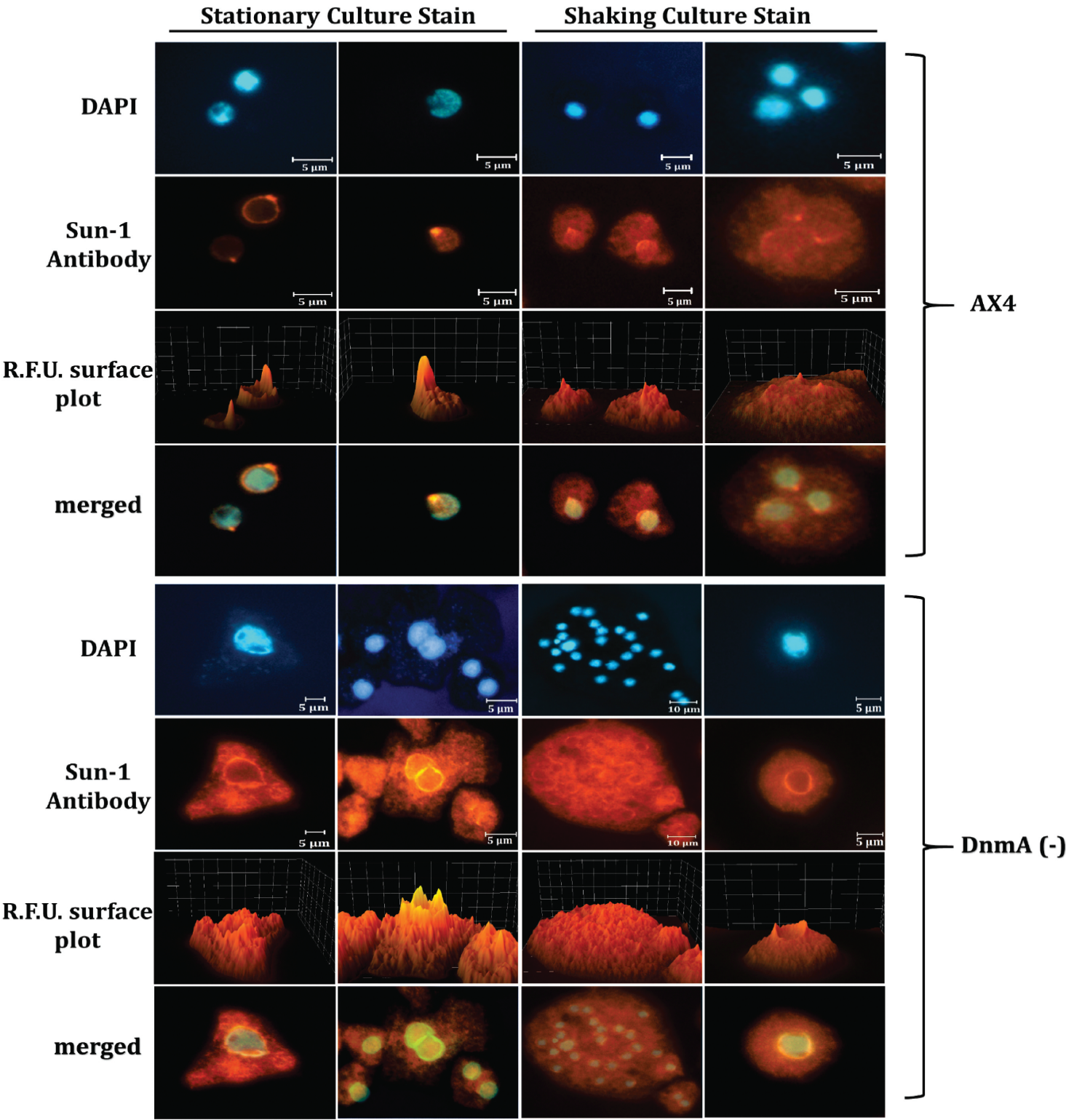

Supplement: jkaf152_Supplementary_Data [file jkaf152_supplementary_data.zip › Figure_S3_G3-2025-406015.pdf]

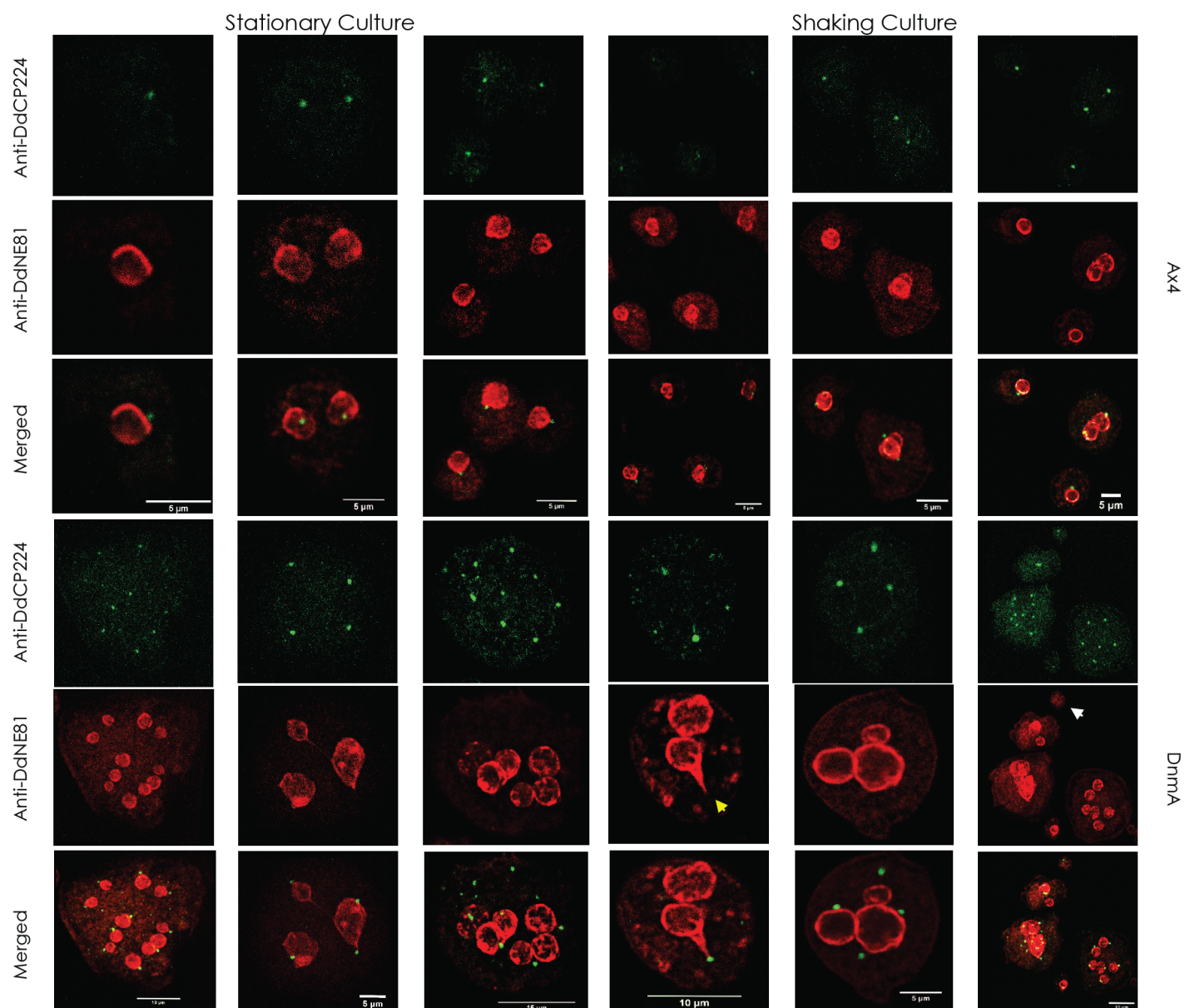

Supplement: jkaf152_Supplementary_Data [file jkaf152_supplementary_data.zip › Figure_S4_G3-2025-406015.pdf]

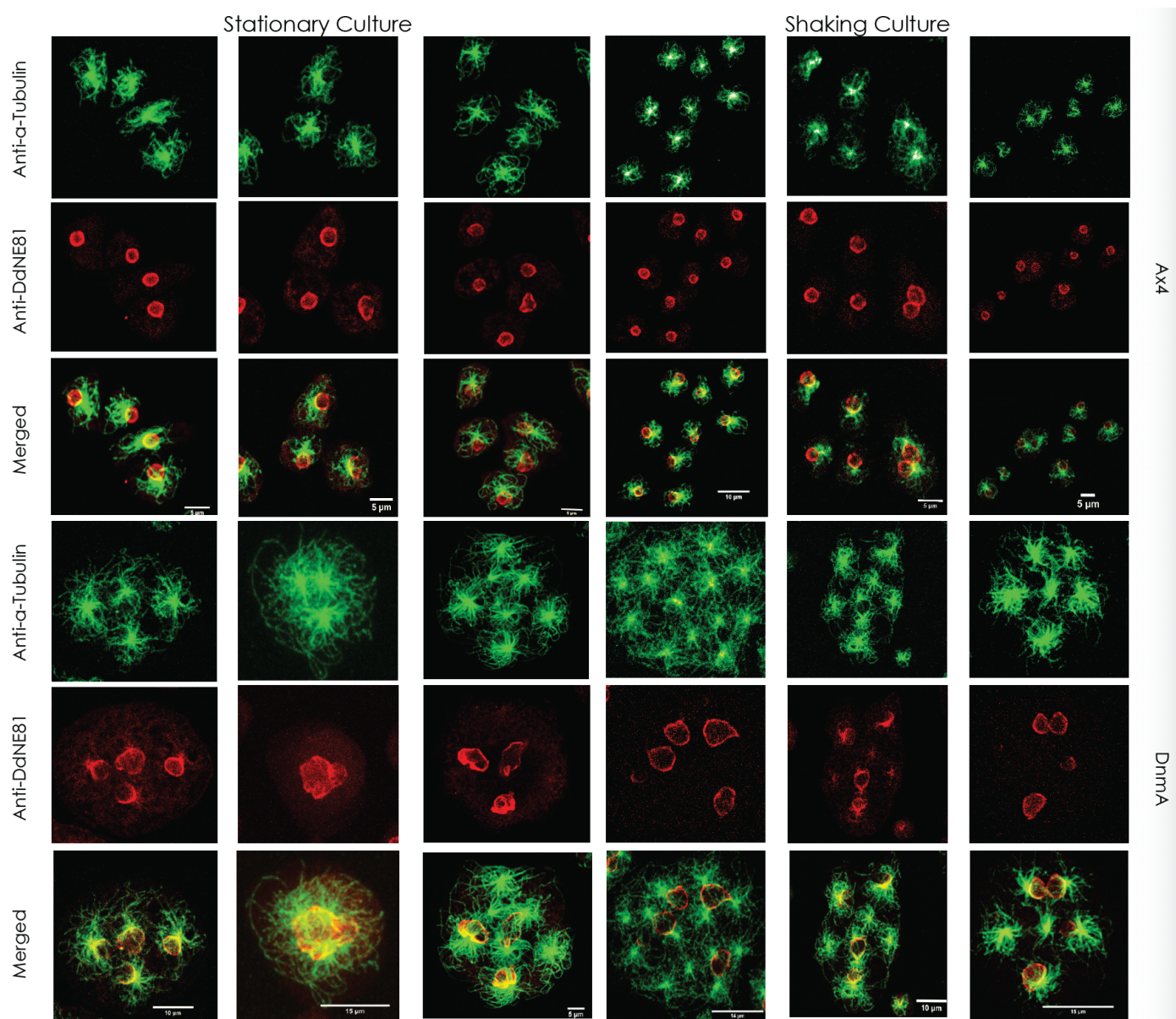

Supplement: jkaf152_Supplementary_Data [file jkaf152_supplementary_data.zip › Figure_S5_G3-2025-406015.pdf]

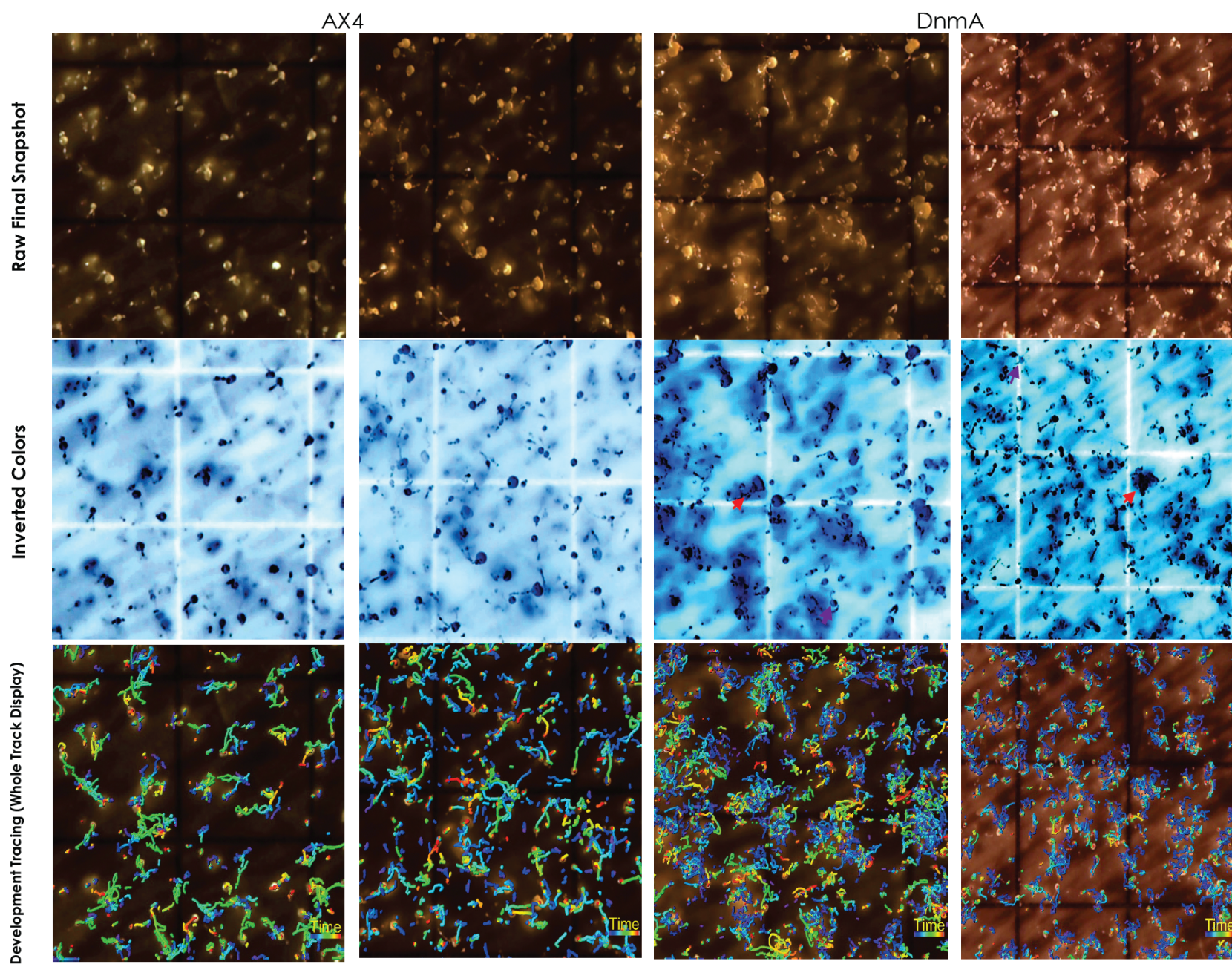

Supplement: jkaf152_Supplementary_Data [file jkaf152_supplementary_data.zip › Figure_S6_G3-2025-406015.pdf]

**A**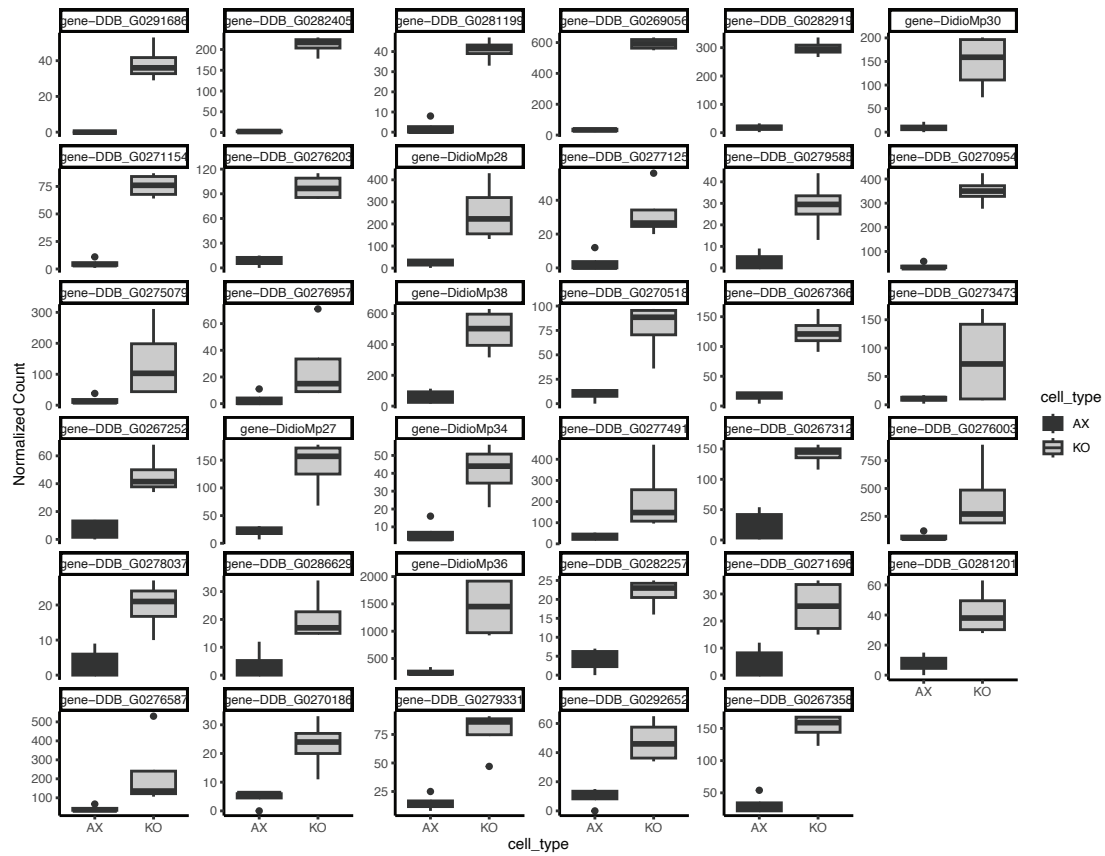**B**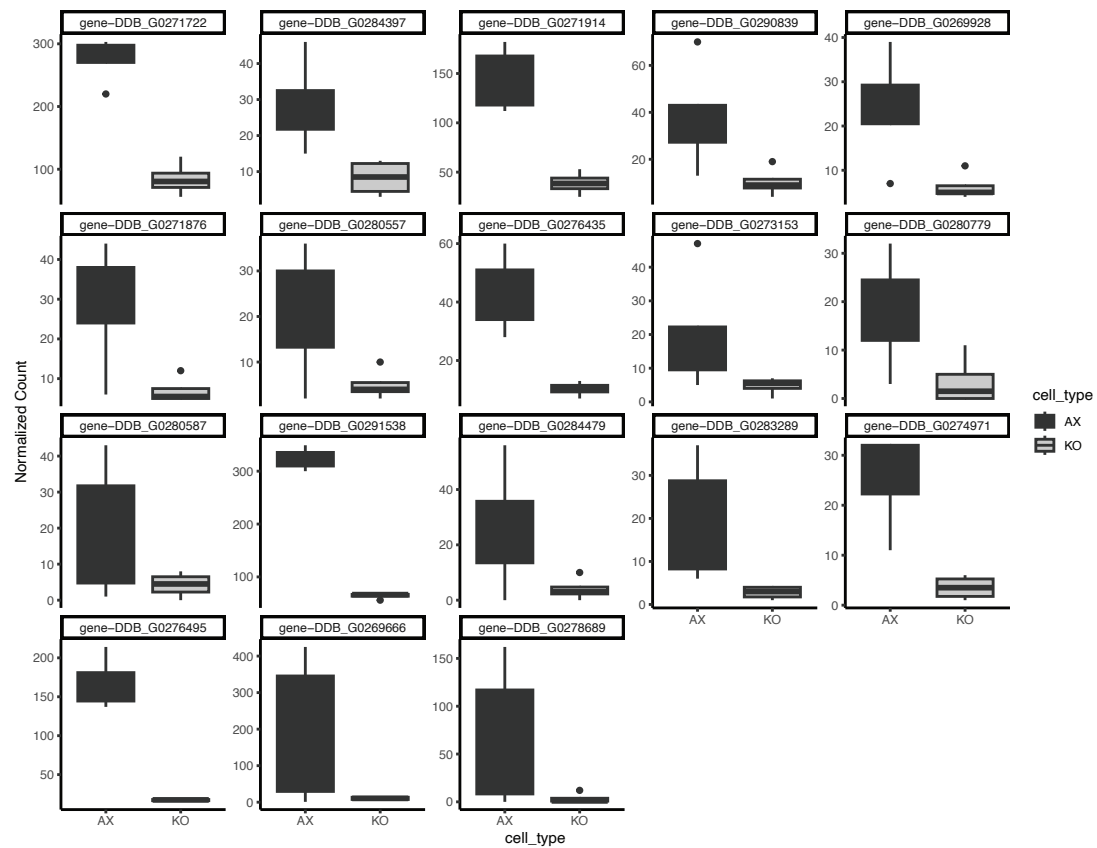

Supplement: jkaf152_Supplementary_Data [file jkaf152_supplementary_data.zip › Figure_S7_G3-2025-406015.pdf]

**A**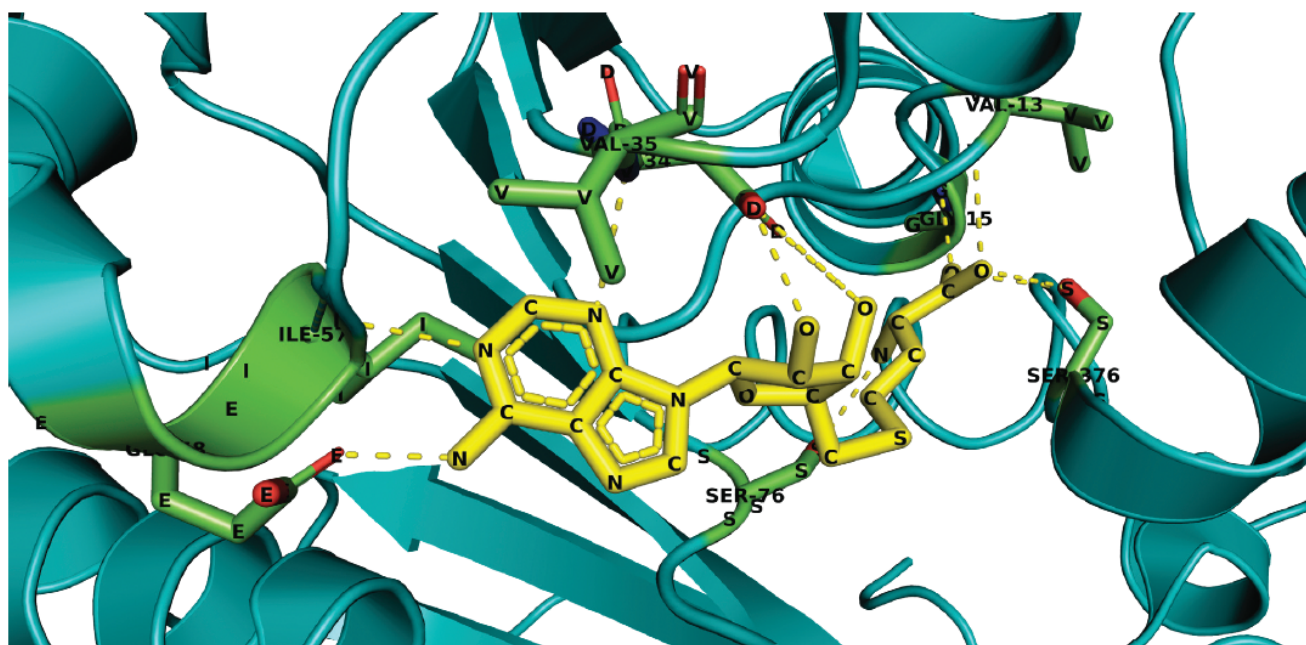**B**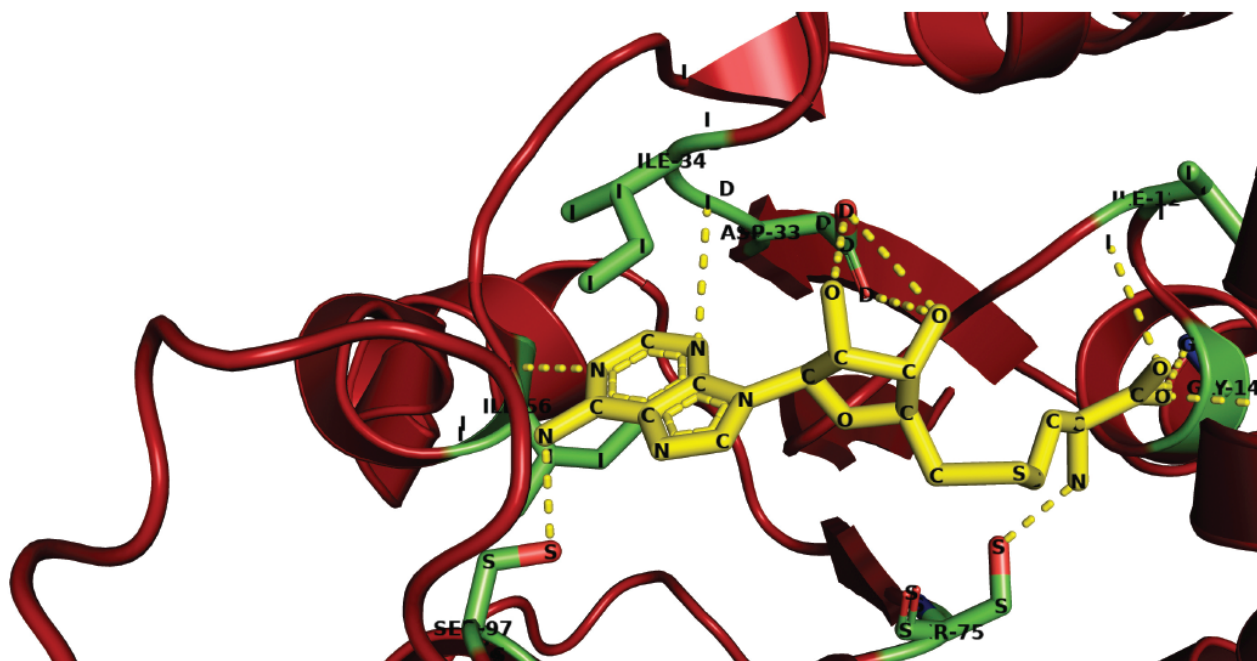

Supplement: jkaf152_Supplementary_Data [file jkaf152_supplementary_data.zip › Figure_S8_G3-2025-406015.pdf]

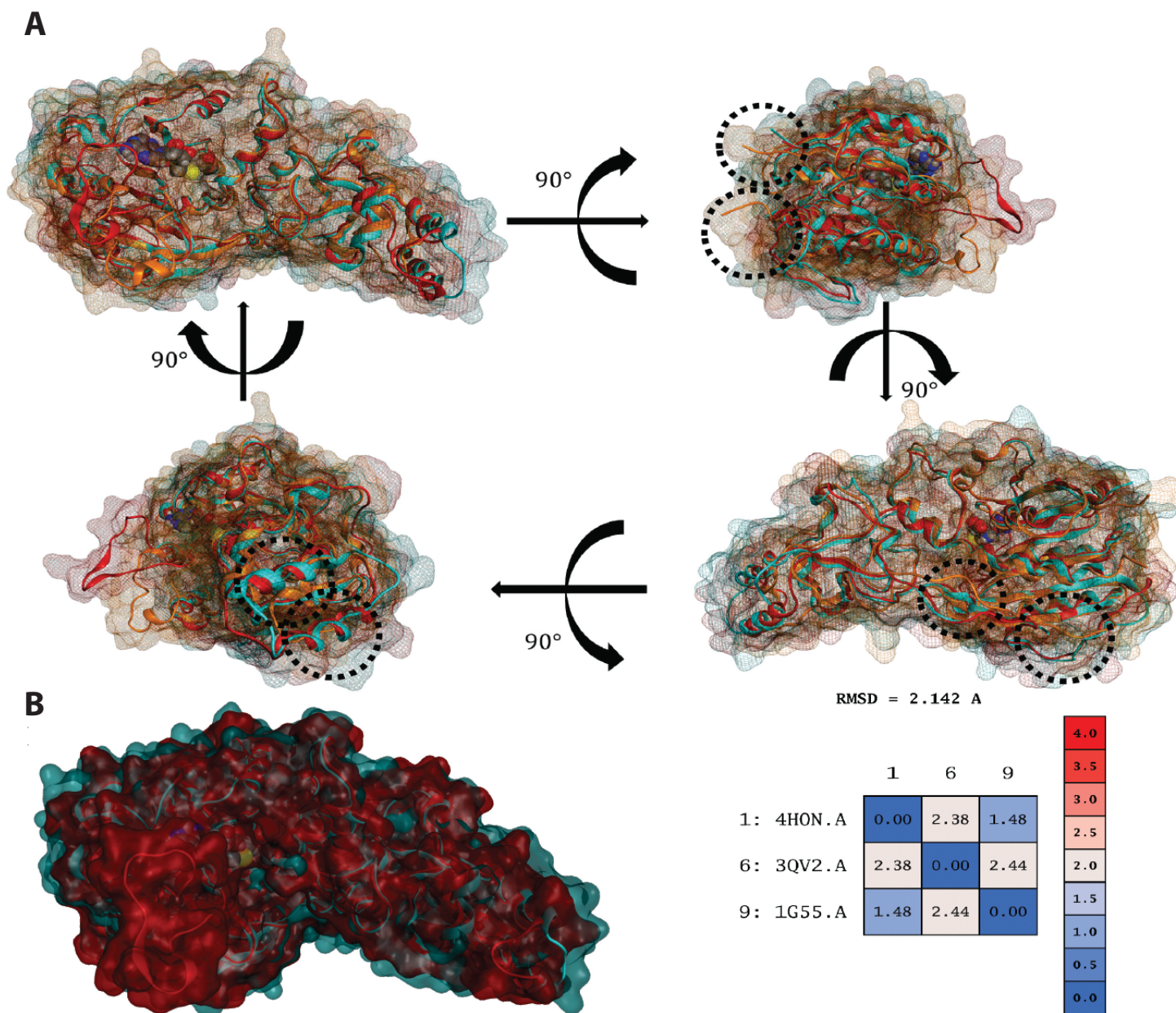

Supplement: jkaf152_Supplementary_Data [file jkaf152_supplementary_data.zip › Figure_S9_G3-2025-406015.pdf]
